# Public willingness to participate in personalized health research and biobanking: A large-scale Swiss survey

**DOI:** 10.1371/journal.pone.0249141

**Published:** 2021-04-01

**Authors:** Caroline Brall, Claudia Berlin, Marcel Zwahlen, Kelly E. Ormond, Matthias Egger, Effy Vayena

**Affiliations:** 1 Health Ethics and Policy Lab, Department of Health Sciences and Technology, ETH Zurich, Zurich, Switzerland; 2 Institute for Social and Preventive Medicine, University of Bern, Bern, Switzerland; 3 Department of Genetics and Stanford Center for Biomedical Ethics, Stanford University School of Medicine, Stanford, California, United States of America; University of Oxford, UNITED KINGDOM

## Abstract

This paper reports survey findings on the Swiss public’s willingness, attitudes, and concerns regarding personalized health research participation by providing health information and biological material. The survey reached a sample of 15,106 Swiss residents, from which we received 5,156 responses (34.1% response rate). The majority of respondents were aware of research using human biological samples (71.0%) and held a positive opinion towards this type of research (62.4%). Of all respondents, 53.6% indicated that they would be willing to participate in a personalized health research project. Willingness to participate was higher in younger, higher educated, non-religious respondents with a background in the health sector. Respondents were more willing to provide ‘traditional’ types of health data, such as health questionnaires, blood or biological samples, as opposed to social media or app-related data. All respondents valued the return of individual research results, including risk for diseases for which no treatment is available. Our findings highlight that alongside general positive attitudes towards personalized health research using data and samples, respondents have concerns about data privacy and re-use. Concerns included potential discrimination, confidentiality breaches, and misuse of data for commercial or marketing purposes. The findings of this large-scale survey can inform Swiss research institutions and assist policymakers with adjusting practices and developing policies to better meet the needs and preferences of the public. Efforts in this direction could focus on research initiatives engaging in transparent communication, education, and engagement activities, to increase public understanding and insight into data sharing activities, and ultimately strengthen personalized health research efforts.

## Introduction

Personalized health research has received increasing attention and support over recent years in Switzerland. The European Commission defines personalized health as developing a “medical model using characterization of individuals’ phenotypes and genotypes (e.g. molecular profiling, medical imaging, lifestyle data) for tailoring the right therapeutic strategy for the right person at the right time and/or to determine the predisposition to disease and/or to deliver timely and targeted prevention” [1]. As the latter description suggests, this research depends on the willing contribution of large amounts of health data and genetic information by individuals [2]. Yet, the extent to which the Swiss public today concurs or disagrees with research biobanks using genetic and health data remains unknown. Established Swiss cohorts are the Swiss HIV cohort, a systematic longitudinal study enrolling more than 20’000 HIV-infected individuals in Switzerland, which was established in 1988 and SAPALDIA (Study on Air Pollution And Lung Disease In Adults), which investigates the effects of life style and environment on chronic diseases and aging in 10’000 adults of the Swiss general population since 1991. In terms of biobanks, 37 biobanks and biobank infrastructures are registered in the Swiss Biobanking Platform. They are mainly affiliated to the five Swiss university hospitals. To pool available data and to foster genetic research through a nationally coordinated data infrastructure, the federal government established the Swiss Personalized Health Network (SPHN) in 2017.

What little we do know about the Swiss public’s perceptions of personalized health to date comes from a public survey mandated by the Swiss Federal Office of Public Health in 2018, as well as studies involving patients receiving medical treatment [3, 4]. The former survey of 1,983 Swiss residents revealed that citizens had limited knowledge about research with humans and its legalities in Switzerland. Interestingly, despite being generally uninformed, approximately half of respondents were hypothetically willing to provide their data in the form of questionnaires or biological samples for human research purposes [4]. Similarly, a study assessing the consent of 25,000 hospital patients revealed that 79% were willing to provide personal data and blood samples for research [4]. These studies help paint a picture of Swiss public perspectives, yet do not depict a representative overview. To support governmental efforts and promote personalized health research and its future developments in Switzerland, we need to better understand what motivates and concerns potential contributors of data and biological samples, and identify what they expect from personalized health research infrastructures. We can then address these needs when building or expanding biobanks and establishing personalized health research cohorts. To meet this need, we invited a representative sample of 15,106 Swiss residents to respond to our survey. Our goal was to identify motives, concerns, and expectations of the Swiss public about providing health information and biological material for personalized health research.

This paper presents findings from a survey about the Swiss public’s willingness, attitudes, and concerns regarding participation in personalized health research. First, we report on the demographic profiles of survey participants and their willingness to donate health information and biological material for personalized health research. Second, we present participant opinions towards research with human biological samples and preferences for providing certain data types. Third, we outline concerns about participation in personalized health research and which kinds of results participants prefer to receive.

## Methods

### Sample

We conducted a cross-sectional survey with a potential sample of 15,106 individuals in Switzerland to explore attitudes, concerns, and expectations towards the hypothetical provision of health and genetic data for personalized health research. The size of the sampling frame was determined according to power analyses, assuming a response rate of 25%. Participants had to be over 18 years of age and reside in Switzerland. The Swiss Federal Statistical Office (FSO) provided the stratified random sample which covered gender, four age groups (between 18 and 64+), and the three main language regions (German, French, Italian) across all geographical regions in Switzerland. The minority language regions (French, Italian) were oversampled. Although 62% of the Swiss population speak German, 23% French, and 8% Italian [5], sampling accounted for 2:1:1 for German, French and Italian so that a sufficient amount of responses from the French and Italian speaking population could be reached. The legal basis for the provision of samples is Article 13c (2) of the Swiss Statistical Survey Ordinance (SR 431.012.1).

Participants were notified of the survey by regular mail in their language of correspondence, which they indicated upon registration at their municipality. They first received a letter briefly describing the scope and background of the study, with instructions to complete the survey online using a dedicated web platform available in German, French, Italian, and English (Qualtrics). The letter also provided participants with a secure individualized login password, which enabled anonymous tracking of individual responses. We then sent two reminders to non-responders after three and seven weeks. The paper-based version of the questionnaire was also attached in the second reminder (in the indicated language of correspondence), with a pre-paid envelope for returning the questionnaire to the research team. The paper-based questionnaires also contained unique personal codes that matched participants’ answers with their sociodemographic characteristics, as provided by the FSO. If a participant filled out both versions, we only included the online version.

By completing the questionnaire, participants provided their informed consent. We collected answers for 20 weeks (16^th^ September 2019 until 31^st^ January 2020) until responses abridged. The project complies with data protection regulations at each research institution (ETH Zurich and the University of Bern). The Ethics Committee of ETH Zurich (EK 2018-N-66) approved the study.

### Questionnaire development and pretesting

**Fig 1:**
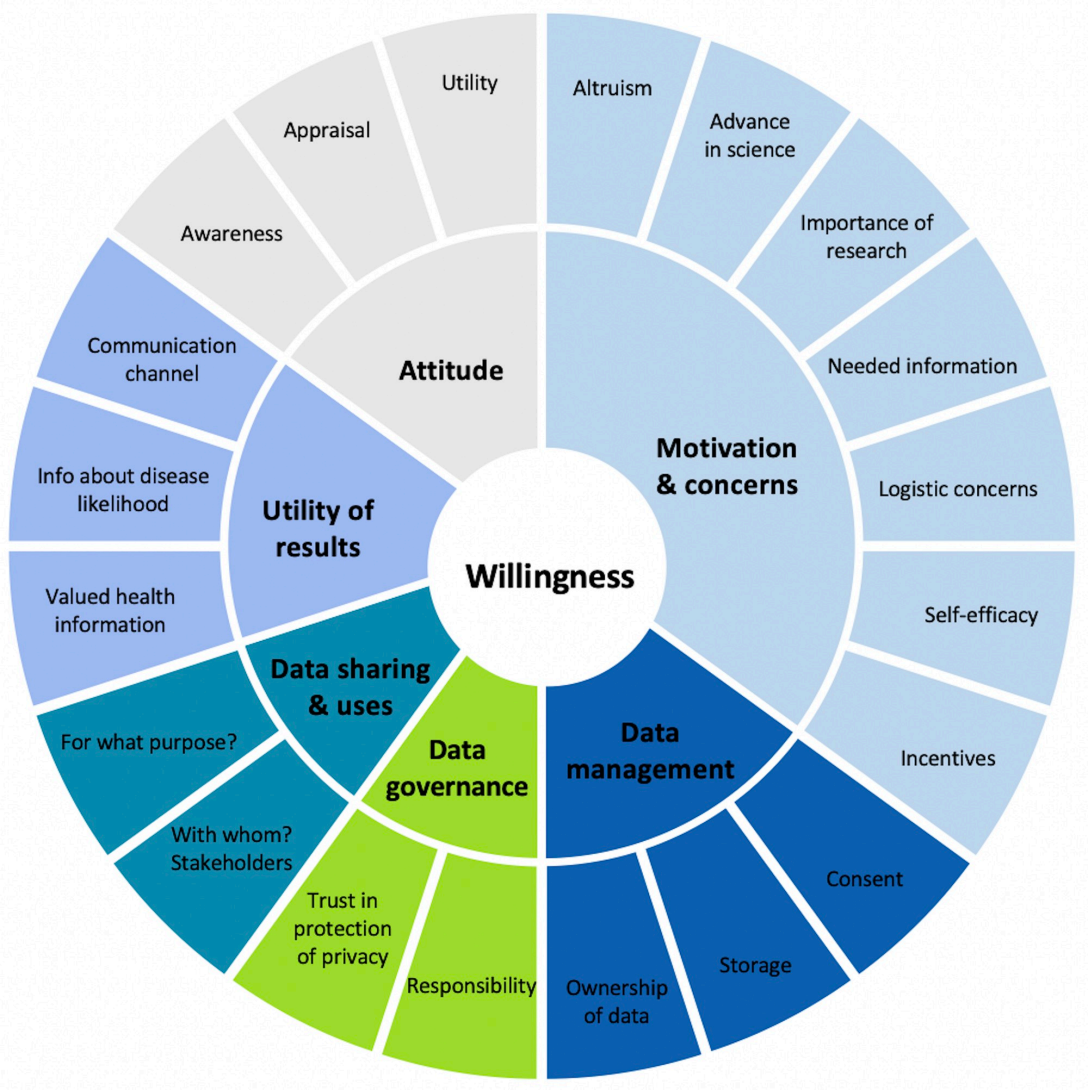
Individual’s willingness to participate in personalized health research—A conceptual framework.

Based on a narrative literature review of scholarly articles on donating health data for research, we developed a conceptual framework (Fig 1) that delineates six underlying concepts of individuals’ willingness to donate health data and biological material for research purposes. Identified categories included general attitude, motivations and concerns, as well as expectations towards data management, data governance, data sharing and uses, and willingness to receive results. Although the survey explored all six categories, this paper focuses on three which capture the general expectations towards donating data and biological material for personalised health research: attitudes, concerns, and willingness to receive results.

Based on the conceptual framework, we drafted and refined an English language questionnaire. The outcome consisted of 23 closed (binary and 5-point Likert scale) and multiple-choice items (see supplementary material). For some questions, the answer options “I don’t know” and/or “other” were provided. In the online survey, questions could not be skipped. We then translated the questionnaire from English into the three main languages in Switzerland: German, French, and Italian. Each translation was revised and checked by two native speakers to ensure the highest level of concept-matching and language accuracy. To ensure readability, construct, and content validity, we conducted a two-wave pilot test: first an expert wave and second a convenience sample wave. The first wave involved 14 experts in the field of bioethics and health sciences. Nine provided written feedback, and five filled out the questionnaire and commented in a cognitive interview. Results of this expert wave helped refine and improve the clarity, stringency, and exhaustiveness of the survey questions. The research team then conducted pretesting with the convenience sample to evaluate readability and comprehension among the target population. The expert sample from the first wave identified the participants for this step by applying snowball sampling in their social networks. As a result, the individuals selected represented different age and gender groups, and divergent geographical, language, educational, and socioeconomic backgrounds. Data collection for the pilot test continued until data saturation was achieved at 17 responses. Based on the latter results, we then revised and updated the survey translations.

### Statistical analysis

We analysed the data using the software STATA (version 15, College Station, TX, USA). Before analysis, we evaluated data for completeness and normal distribution of continuous variables. We included only questionnaires with a minimum of 50% data completeness. The research team matched the survey data with participants’ demographics through a unique identifier. The demographic data linked from FSO data included gender, age, language, household size, nationality, marital status, and municipality of residence. In a next step, we calculated and applied survey weights (using gender, age, language region) to account for differences between respondents and the general population of Switzerland. For obtaining relative proportions, we used modified multivariable Poisson regression to adjust for confounders and identify factors independently associated with willingness to provide health data and biological samples for personalized health research [6]. We used relative proportions because they enable a more straightforward interpretation compared to odds ratios for instance. For example, comparing a proportion of 60% to one of 30% results in a relative proportion of 2.0, but an odds ratio of 3.5. In addition to the variables provided by the FSO, we included the type of response (online vs. paper-based) and the self-reported variables of biological children, education, religious views, working in the health sector, and health status in the analyses. Thus our analysis included the variables: age, gender, nationality, number of household members, marital status, having biological children, language region, residence in a urban/rural municipality, education, religiousity, currently or previously working in the health sector, health status, and the type of survey response.

## Results

### Sample description

We received a total of 5,156 responses, representing an overall response rate of 34.1%. We received 950 responses after the initial invite, 2,834 after the first reminder and 1,372 after the second reminder. The majority, 3,519 (68%), were web-based responses. We deleted 70 responses: 63 which were paper-based with less than 50% answers completed, four duplicates completed as both online and paper-based, and three paper-based questionnaires with missing identifiers. The final analysis thus contained 5,086 complete responses (a 33.7% response rate after excluding incomplete questionnaires). S1 Table portrays the key demographic characteristics of respondents and non-respondents. The percentage of the following characteristics differed between the responder and non-responder group: Swiss nationality (respondents: 82.9% vs. non-respondents: 71.8% vs. total sample: 75.6%), Non-Swiss nationality (17.1% vs. 28.2% vs. 24.4%), one household member (15% vs. 19.5% vs. 17.9%), single persons (32.6% vs. 40.7% vs. 37.9%) as well as married persons (53.9% vs. 45.6% vs. 48.4%). Table 1 presents an overview of the distribution of respondents by sociodemographic characteristics, the proportion of respondents willing to participate in a health research project, and results of the adjusted model using modified Poisson regression (all weighted proportions except n).

**Table 1:** Willingness to participate in personalized health research by providing health data and biological samples according to sociodemographic factors (weighted proportions).

	Sample	Population		Proportion willing to participate & provide health data & samples		Adjusted Relative Proportion (RP)	
	n	%	95% CI	%	95% CI	adjusted RP	95% CI
**Total**	**5,086**	**100**		**53.57**	**[51.86,55.26]**		
**Age group**						**p = 0.0167**	
18–24	594	8.63	[7.84,9.49]	60.74	[55.87,65.42]	1.29	[1.12,1.49]
25–34	758	17.65	[16.32,19.07]	59.93	[55.59,64.12]	1.16	[1.03,1.31]
35–44	632	18.51	[17.05,20.07]	56.51	[51.83,61.08]	1.12	[1.00,1.26]
45–54	927	19.48	[18.18,20.86]	48.88	[45.06,52.72]	1	
55–64	1,005	17.46	[16.28,18.71]	51.94	[48.14,55.71]	1.04	[0.93,1.16]
65–74	857	13.1	[12.14,14.12]	48.63	[44.70,52.58]	0.98	[0.87,1.11]
75–79	313	5.16	[4.53,5.87]	44.41	[37.92,51.09]	0.91	[0.77,1.09]
Total	5,086	100		53.57	[51.86,55.26]		
**Sex**						**p = 0.8628**	
Male	2,451	50.09	[48.39,51.79]	53.47	[50.99,55.94]	1	
Female	2,635	49.91	[48.21,51.61]	53.66	[51.32,55.99]	1.01	[0.94,1.07]
Total	5,086	100		53.57	[51.86,55.26]		
**Nationality**						**p = 0.0404**	
Swiss	4,216	76.03	[74.35,77.62]	54.69	[52.87,56.51]	1	
Non-Swiss	870	23.97	[22.38,25.65]	49.99	[45.88,54.10]	0.91	[0.83,1.00]
Total	5,086	100		53.57	[51.86,55.26]		
**Number of household members**						**p = 0.0355**	
1	760	18.12	[16.73,19.58]	56.16	[51.70,60.53]	1	
2	1,854	35.47	[33.89,37.09]	54.06	[51.30,56.80]	1	[0.91,1.11]
3–5	2,347	43.65	[41.99,45.34]	52.82	[50.30,55.33]	0.91	[0.81,1.02]
6 persons and more	125	2.75	[2.24,3.39]	42.13	[32.20,52.73]	0.76	[0.59,0.99]
Total	5,086	100		53.57	[51.86,55.26]		
**Marital status**						**p = 0.3284**	
single	1,669	35.25	[33.59,36.94]	59.46	[56.43,62.42]	1	
married	2,733	50.93	[49.23,52.64]	49.38	[47.09,51.67]	0.96	[0.86,1.07]
widowed	150	3.1	[2.58,3.73]	50.72	[41.35,60.05]	1.09	[0.87,1.36]
divorced	534	10.72	[9.73,11.80]	54.8	[49.66,59.83]	1.05	[0.92,1.20]
Total	5,086	100		53.57	[51.86,55.26]		
**Biological children**						**p = 0.2843**	
Yes	2,968	57.62	[55.91,59.32]	50.82	[48.63,53.00]	1	
No	2,074	42.38	[40.68,44.09]	57.62	[54.91,60.30]	0.95	[0.87,1.04]
Missing	44						
Total	5,086	100		53.7	[51.99,55.40]		
**Language region**						**p = 0.0168**	
German	2,257	70.76	[70.30,71.23]	54.33	[52.14,56.51]	1	
French	1,366	24.51	[24.06,24.97]	51.02	[48.16,53.88]	0.93	[0.87,1.00]
Italian	1,463	4.72	[4.63,4.81]	55.25	[52.54,57.92]	1.03	[0.97,1.10]
Total	5,086	100		53.57	[51.86,55.26]		
**Urban/rural municipality**						**p = 0.2382**	
urban	3,104	61.04	[59.39,62.67]	54.07	[51.86,56.27]	1	
intermediary	1,086	21.64	[20.30,23.05]	54.51	[50.93,58.05]	1.07	[0.99,1.15]
rural	896	17.31	[16.09,18.60]	50.57	[46.59,54.55]	1.01	[0.92,1.10]
Total	5,086	100		53.57	[51.86,55.26]		
**Education**						**p = 0.0000**	
Compulsory education or less	385	8.36	[7.44,9.37]	30.32	[24.96,36.27]	0.66	[0.55,0.80]
Upper secondary education	3,394	65.23	[63.57,66.86]	51.73	[49.65,53.80]	1	
Tertiary education	1,283	26.41	[24.90,27.98]	65.52	[62.19,68.71]	1.24	[1.16,1.33]
Missing	24						
Total	5,086	100		53.61	[51.90,55.31]		
**Religion**						**p = 0.0023**	
Very much	695	13	[11.92,14.17]	45.58	[41.00,50.24]	1	
Somewhat	2,217	43.55	[41.87,45.25]	50.43	[47.84,53.01]	1.06	[0.94,1.18]
Not at all	2,138	43.45	[41.76,45.15]	59.37	[56.78,61.91]	1.17	[1.04,1.30]
Missing	36						
Total	5,086	100		53.69	[51.98,55.39]		
**Working in health sector?**						**p = 0.0001**	
Yes	1,010	20.17	[18.83,21.57]	62.15	[58.37,65.78]	1	
No	4,063	79.83	[78.43,81.17]	51.39	[49.47,53.29]	0.86	[0.80,0.93]
Missing	13						
Total	5,086	100		53.55	[51.85,55.25]		
**Health status**						**p = 0.5630**	
Very unhealthy	61	0.76	[0.53,1.10]	49.65	[32.40,66.99]	1	
Somewhat unhealthy	115	2.02	[1.62,2.52]	47.4	[36.44,58.61]	1.05	[0.68,1.61]
Neutral	661	13.51	[12.38,14.73]	52.05	[47.36,56.70]	1.04	[0.72,1.51]
Somewhat healthy	2,578	47.13	[45.45,48.82]	53.39	[50.95,55.81]	1	[0.70,1.44]
Very healthy	1,643	36.57	[34.93,38.24]	55.18	[52.28,58.05]	0.96	[0.67,1.38]
Missing	28						
Total	5,086	100		53.72	[52.01,55.42]		
**Type of response**						**p = 0.0000**	
Web-based	3,519	71.04	[69.50,72.54]	57.03	[55.00,59.03]	1	
Paper-based	1,567	28.96	[27.46,30.50]	44.87	[41.79,48.00]	0.83	[0.77,0.90]
Total	5,086	100		53.57	[51.86,55.26]		

### Willingness to provide health data and biological samples

More than half of respondents (53.6%) indicated a willingness to participate in a hypothetical personalized health research study by providing health data and/or biological samples. The analysis showed willingness varied with sociodemographic characteristics (Table 1). The biggest differences were found in age (18–24 years: 60.7%, 75–79 years: 44.4%), education (compulsory education or less: 30.3%, tertiary education: 65.5%), self-reported religiosity (very much: 45.6%, not at all: 59.4%), number of household members (1 person: 56.2%, 6 persons and more: 42.1%), working in the health sector (yes: 62.2%, no: 51.4%), marital status (single: 59.5%, married: 49.4%), having biological children (yes: 50.8%, no: 57.6%), health status (very unhealthy: 49.7%, very healthy: 55.2%) and nationality (Swiss: 54.7%, Non-Swiss: 50%).

Based on the multivariable analysis (Table 1), we found the younger age groups more willing to participate (18–24: adjusted Relative Proportions (aRP) = 1.29, 95% CI: 1.12–1.49; 25–34: aRP = 1.16, 95% CI: 1.03–1.31) compared to older age groups (55–64; 65–74; 75–79). Persons without Swiss nationality, from the French language region, and living in large households (6 persons and more) were found to be less willing to provide health data and biological samples. We found level of education to have the strongest association with willingness to participate. Compared to respondents with upper secondary education, those with compulsory education or less were less willing (aRP = 0.66, 95% CI: 0.55–0.80) whereas people with tertiary education were more willing (aRP = 1.24, 95% CI: 1.16–1.33) to participate. Furthermore, we found the relative proportion of willingness to participate in a health research project higher for non-religious persons or those who currently or previously worked in the health sector.

Regarding type of response, 57.0% of the web-based responses indicated a willingness to participate, compared with 44.9% willingness by paper-based respondents. The sociodemographic characteristics between these sub-samples show that respondents using the paper-based questionnaire were often older, female, living in smaller households, not single, had biological children, and had lower levels of education (see S2 Table). However, analysis of the responses as a whole revealed that except for having biological children, the factors of gender, marital status, health status, or residence in an urban/rural municipality did not impact respondents’ willingness to participate. In addition, after adjusting for multiple characteristics in the Poisson regression analysis, the association between lower willingness to participate and persons who responded via paper-based questionnaire persisted (aRP = 0.83, 95% CI: 0.77–0.90).

### Awareness and opinion towards research with human biological samples

**Fig 2:**
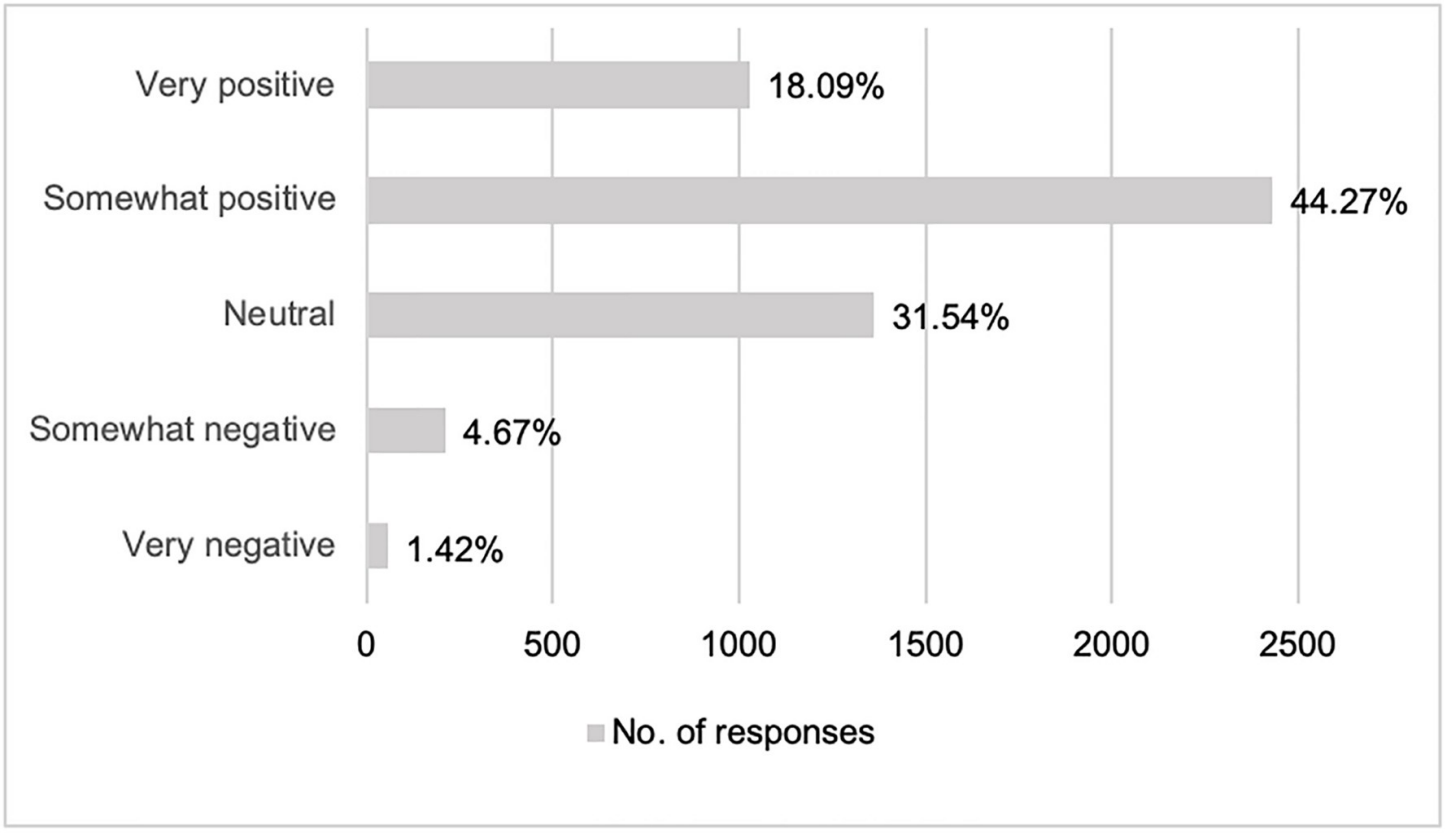
Opinion towards research with human biological samples.

The majority of respondents (71%) reported awareness of research using human biological samples. As shown by Fig 2, a majority indicated a rather positive attitude towards such research, with responses of “somewhat positive” (44.27%, n = 2,424) or “very positive” (18.09%, n = 1,021). Only 4.67% indicated having a “somewhat negative” opinion (n = 209) and 1.42% a “very negative” opinion (n = 53). The analysis revealed that willingness to participate in personalized health research was 11 times more likely in respondents reporting an opinion of “very positive” compared to “very negative” [aRP = 11.22, 95% CI: 4.17–30.21].

### Sensitivity towards different types of data

**Fig 3:**
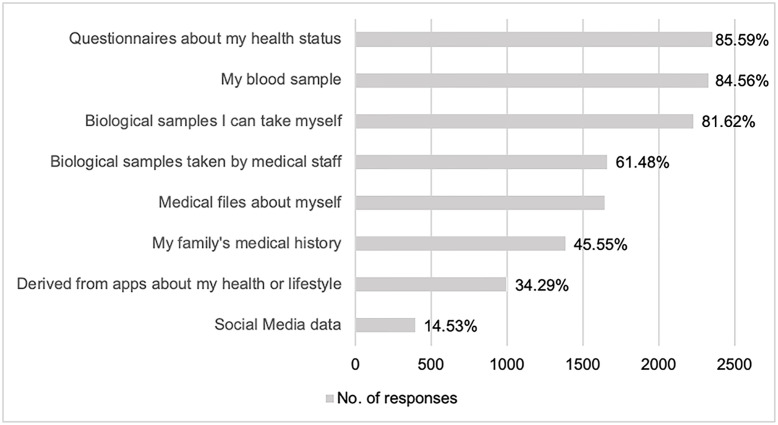
Willingness of respondents to provide specific types of data.

Fig 3 shows the types of data respondents indicated being willing to share. A majority of those who are willing to share their health data and/or biological samples indicated they would like to share questionnaires about their health status (85.59%, n = 2,352), blood samples (84.56%, n = 2,326 respondents), and self-collected biological samples, such as hair, saliva, or urine (81.62%, n = 2,224 respondents). Only a few respondents were willing to share data derived from apps about their health or lifestyle (such as heart rates, exercise trackers, or food logs; 34.29%, n = 994) or their social media data (14.53%, n = 392).

### Concerns about participation

**Fig 4:**
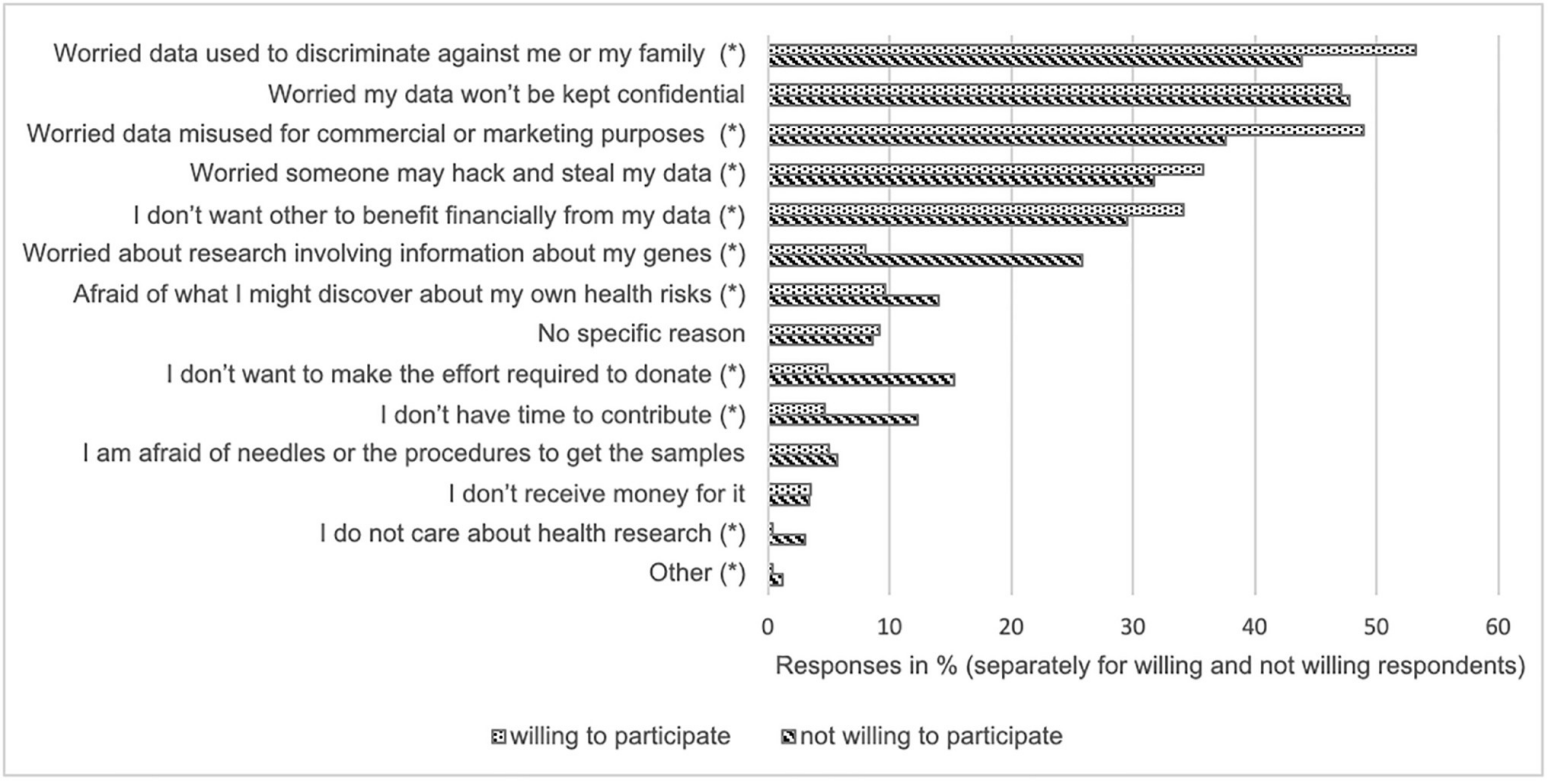
Overview of concerns for persons willing and not willing to participate in a personalized health research project by providing health data and biological samples. *Indicates statistical difference between these two groups, p<0.05.

Respondents expressed several concerns about participating in a research project that would use their health data and/or biological samples. As shown in Fig 4, participants could choose up to three concerns most relevant to them. Out of all respondents (5,025 persons, 13,371 answers in total), most were worried that participation could lead to discrimination against them or their family (46.8%). This concern was closely followed by the concern that their data would not be kept confidential (46.3%) or would be misused for commercial or marketing purposes (45.5%). Additional frequent concerns were that someone might hack and steal their data (31.9%) and that others would benefit financially from their data (31.2%). These five most frequent concerns were consistent whether or not respondents were willing to participate in a personalized health research project. However in 10 of the 14 response options we discovered differences between these two groups (Fig 4). Individuals not willing to participate were less concerned that the data would be used to discriminate against them and their family (willing: 53.2%, not willing: 43.8%) or would be misused for commercial or marketing purposes (willing: 48.9%, not willing: 37.6%), but were more concerned about research involving genetic information (willing: 8.1%, not willing: 25.9%).

### The willingness to receive results

Table 2 reports the types of research results that participants would hypothetically like to receive if participating in a personalized health research study. Respondents were generally interested in receiving results, however preferences differed slightly. A large majority reported wanting to receive details about their basic medical information, e.g. blood count (83.2% indicated “yes, 10.0% “no”, and 6.8% “don’t know”). Many participants also indicated a wish to receive information about the following; risk for diseases with available medical treatment (e.g. some types of cancer) (73.8%); risk for diseases for which only preventive action can be undertaken (e.g. heart disease) (72.6%); and effects of lifestyle (e.g. smoking, weight etc.) on one’s risk of a medical condition (69.6%).

**Table 2:** Willingness to receive distinct types of research results (weighted proportions).

	Overall			Willing to participate			Not willing to participate		
Type of research results	Yes	No	Don’t know	Yes	No	Don’t know	Yes	No	Don’t know
Basic medical information	83.2	10.0	6.8	90.8	6.4	2.8	74.3	14.2	11.4
Lifestyle affects my risk of getting a medical condition	69.6	22.3	8.2	79.6	16	4.4	58	29.6	12.4
Diseases for which medical treatments are available	73.8	16.1	10.2	83.7	10.2	6.1	62.1	23.1	14.9
Diseases for which only preventive actions can be undertaken	72.6	17.5	10.0	81.9	12.4	5.7	61.7	23.4	14.9
Diseases for which no treatment is available	54.4	30.5	15.1	64	25.1	10.9	43.2	37	19.8
Research results about the study in general	66.2	19.7	14.1	77.9	13.8	8.3	52.8	26.6	20.6

Fewer respondents wished to receive general research results about the study, which do not apply to them individually (66.2%). Fewest respondents chose to receive results about risk of diseases for which no medical treatment is available, but that could impact their well-being or decisions about their career or family planning (e.g. Alzheimer’s disease or dementia) (54.4%). Respondents who indicated their willingness to participate in a personalized health research project are also more interested in receiving individual research results than those who are not willing to participate. Those who are not willing to participate furthermore chose more often the answer option “don’t know”.

## Discussion

Results show that just over half of respondents (53.6%) in this diverse national sample would be willing to participate in a personalized health research project by providing health data and/or biological samples. This finding aligns with results from a 2018 Swiss study, which reported a willingness of 49% [3]. Upon comparison, similar surveys from other countries demonstrate higher rates: with 86% in Italy [7], 86% in Sweden [8], 83.5% in Korea [9], 70.4% in Germany [10]. Others show similar rates: with 56% in Germany [11], and 54% in the US [12]. At the same time, a global survey of 36,268 respondents across 22 countries indicated that willingness to donate data to doctors, and non-profit and for-profit researchers, was generally “low” (47.4%), with variation among countries (from 29% in Japan up to 63.7% in Mexico) [13]. These differences in outcomes imply that results of comparable international studies must be carefully interpreted. Several factors might explain the divergence: whether or not studies occurred in a healthcare setting or not, phrasing of survey questions, and the population surveyed (general population, patients, or research participants), as well as interpretation of outcomes as low or high. For example, the Italian survey sample consisted of family members of geriatric outpatient unit patients [7], who might be potentially more open-minded towards health care and health research. Generalizing or comparing findings across contexts is therefore not always possible. One potential reason why our survey results indicate lower rates of willingness in Switzerland could be the high value placed on autonomy and individual responsibility among the Swiss population [14]. Preserving one’s health would take priority over donating data or samples to health research. In addition, Swiss law treats personal health data and biological samples as sensitive, reinforcing the common understanding that such data must be protected [15].

Our finding that willingness to participate in personalized health research is significantly higher among young (18–24 years), highly educated, and non-religious participants, particularly those with a background in health care, corresponds with similar biobank and research participation studies [12, 16–18]. To compare different points in time, we did not find many similar studies. Yet two studies from 2001 from the US [19] and 2004 from Singapore [20], show that 42% and 49.3% of respondents were willing to donate and store blood for genetic research, indicating no significant differences in the last 20 years. Although age, education, religion, and background in healthcare have been clearly shown to influence willingness to participate in research, actions undertaken in recent years to improve participation, also among unrepresented groups, remain less well reported. Nevertheless, some guidance does exist. For example, running information campaigns designed for different ages and educational levels prior to a research endeavor have been shown to increase enrolment across diverse groups [21]. However, actual participation rates in human and biobank research consistently remain lower than self-reported, hypothetical willingness [22]. This difference could result from logistical and time constraints, or lack of practical information about where and how to participate. Taking these points into consideration and fostering public engagement will be key for achieving higher willingness and participation levels in Switzerland.

In deciding whether or not to participate in a personalized health research study, individuals weigh possible benefits against potential risks [3], balancing concern for privacy loss against openness to giving data [23]. Although previous studies confirm privacy-related concerns [18, 24], they do not explore which concerns are most significant [25]. Our study differs in that it identifies three primary participation concerns for the Swiss public. These are concerns for potential discrimination, breaches of confidentiality, and misuse of data for commercial or marketing purposes. As culture and context influence the public‘s understanding, it’s meaning to them and their preferences surrounding privacy, research institutions should engage in tailored education and public discussion, to increase understanding of this complex, often subjective and emotive issue.

Given this background, it is not surprising that participants were least willing to donate social media and app-related data. One possible explanation is that participants do not perceive these data types as valuable to research as they do not stem from the bouquet of “traditional” health data. Another potential reason is a lack of trust in research using these data types, fueled by recent scandals such as the Cambridge Analytica case where social media data was used for undisclosed secondary purposes [26], or the so-called Emotional Contagion Experiment, in which Facebook manipulated the news feed of nearly 700,000 users without their knowledge, to test its ability to alter emotions [27]. International attention from these scandals may have resulted in greater caution over sharing this type of data, and distrust of tech giants with regard to health-related matters [28]. Given that survey participants explicitly reported concerns about privacy and data misuse as most salient to them (as opposed to logistical barriers, fears about physical or emotional harm, or lack of incentives), it is evident that research institutions have novel challenges to solve.

One approach to rebuilding public confidence in research using non-traditional data is to strengthen regulations that differentiate between voluntarily given or involuntarily observed data–for the current regulatory situation resembles the “Wild West” [29]. At the same time, transparency and public education communicating the benefits and limitations of the use of different data types can be strengthened. For example, research institutions should communicate to data donors which types of data are used, under which conditions they may be shared, and the measures in place to guarantee confidentiality and prevention of data misuse [30]. Communication of current agreements and codes of conduct in the health data ecosystem could also be a way forward. This could promote citizen engagement with data governance while fostering transparency and trust in the use of health data [29].

In terms of return of results to the research participant, our findings demonstrate that the majority of respondents would like to receive various types of general and individual results. In research practice, the return of individual research results to participants has long been avoided. Reasons for this abound and include: that research aims to advance knowledge instead of treating individuals; risks can arise in disclosing non-validated genetic findings; and the return has costs associated. Additionally, the therapeutic misconception describes research subjects’ belief to benefit from some form of medical care through participation in a study [31]. In recent years, however, scientific societies and scholars have argued that researchers have broader responsibilities towards their participants, including a duty to inform participants about results produced in the course of research. In line with this, respondents of our study expressed a wish to receive results about risk of diseases for which no medical treatment is available, but that could impact well-being or decisions about career or family planning (e.g. Alzheimer’s disease). This finding is consistent with other studies, which found that some research participants wish to obtain results which are not clinically actionable [17, 32].

To accommodate participants’ differing preferences and to strengthen trust in research, initiatives such as the American All of Us Research Program among others plan to provide the option to choose which type of results are returned [33]. In Switzerland, the Swiss Personalized Health Network (SPHN) recently published recommendations for ethical and responsible reporting of genetic research findings to participants. These recommendations promote reporting of any findings with medical significance, whether within the scope of the study or secondary findings and hence align with this study’s results [34].

Since we conducted this survey between September 2019 and January 2020, our findings present a unique baseline dataset of Swiss public opinions regarding data provision for personalized health research. The global climate around health data and research has since changed due to the COVID-19 pandemic, with Switzerland’s first case reported in February 2020. In efforts to better understand how this novel virus acts on a molecular and population level, and to tailor public health measures, countries and researchers worldwide are increasingly using data. At the same time, the debate around the use of data has grown. On one hand, many fear the deployment of surveillance through data-driven efforts [35, 36]. On the other hand, others argue that public perception of data will change and open the opportunity for new social contracts, as everyone experiences first-hand the need and value of health data for research [37]. Indeed, it will be interesting to identify whether the COVID-19 pandemic changes the Swiss public’s attitude towards sharing health data or towards privacy-related concerns in any way. We thus recommend a follow-up survey on whether the COVID-19 pandemic changed Swiss citizen’s willingness to provide data for health research, to understand consequences for the future.

A methodological limitation of our study–which is inherent to most surveys–is the self-selection bias of respondents, who agree to answer the survey and are willing to provide their data. Individuals who are more positive towards personalized health research could thus be reflected in our sample. Willingness rates might be lower in reality than our results suggest. Furthermore, this survey captures respondents’ attitudes and opinions at a single point in time. A longitudinal survey study could more thoroughly assess how respondents’ views develop and change. As a future direction for research, qualitative studies on what influences citizens’ willingness to provide data and/or samples for personalized health research could yield deeper insights beyond those possible in a survey design. Nonetheless, a strength of our study is a diverse sample representing Swiss residents of all sociodemographic characteristics, regions, and languages spoken. Compared to similar studies, the response rate of our survey is acceptable to fairly high (e.g. 2.9% in Korea [9], 20.4% in Germany [10], and 54% in the US, which however surveyed a panel of persons who have registered at an online survey firm [12]). By weighting the results, we accounted for the variation in response rates across age, gender and language groups. However, we cannot exclude that respondents’ attitudes (within these subpopulations) differ from those who did not respond. Nevertheless, the correction for any effects of age, gender, and language will have attenuated non-response bias. We believe that the weighted analysis results reflect the attitudes and willingness to participate in personalized health research studies of the Swiss population. Therefore, we deem the results to be broadly generalizable to the Swiss population. As respondents had the choice between the web-based or paper-based questionnaire, our survey also represents those less digitally literate or willing to use digital tools.

## Conclusion

Knowing what drives decisions to join personalized health research projects and biobanks is crucial to establishing and maintaining such endeavours successfully. This study presents insights into the Swiss public’s willingness to participate in personalized health research by providing data and/or biological samples. It highlights that personalized health research is supported by slightly more than half of the Swiss public, but that concerns about discrimination, confidentiality and misuse of data for commercial or marketing purposes exist and need to be addressed. The findings can inform Swiss research institutions and policymakers, to adjust practices and develop policies to better meet the needs and preferences of the public. We conclude that besides the implementation transparent communication on the part of research initiatives, tailored public discussions, education, and engagement activities for potential participants are needed, to increase insights into data sharing activities and enable informed choices.

## Supporting information

S1 Table Respondents and non-respondents. (DOCX)

S2 Table Socio-demographic characteristics displayed for chosen type of response. Not weighted.
(DOCX)

S1 File Frequency tables. (DOCX)

S2 File Questionnaire English. (PDF)

S3 File Questionnaire German. (PDF)

S4 File Questionnaire French. (PDF)

S5 File Questionnaire Italian. (PDF)

S6 File Invitation letter German. (PDF)

S7 File Invitation letter French. (PDF)

S8 File Invitation letter Italian. (PDF)

S9 File First reminder German. (PDF)

S10 File First reminder French. (PDF)

S11 File First reminder Italian. (PDF)

S12 File Second reminder German. (PDF)

S13 File Second reminder French. (PDF)

S14 File Second reminder Italian. (PDF)
