# Supplementary material for: Public willingness to participate in personalized health research and biobanking: A large-scale Swiss survey
Source: PLoS One. 2021 Apr 1;16(4):e0249141. doi: 10.1371/journal.pone.0249141 (PMC8016315; doi:10.1371/journal.pone.0249141)
Supplement: S1 Table — (DOCX) [file pone.0249141.s001.docx]

**S1 Table. Respondents and non-respondents.**

|  | **Response to survey** | | | | | | | | |
| --- | --- | --- | --- | --- | --- | --- | --- | --- | --- |
|  | **Yes** | | | **No** | | | **Total** | | |
|  | No. | Col % | Row % | No. | Col % | Row % | No. | Col % | Row % |
| **Age group** |  |  |  |  |  |  |  |  |  |
| 18 - 24 | 595 | 11.6 | 30.7 | 1,346 | 13.5 | 69.3 | 1,941 | 12.8 | 100 |
| 25 - 34 | 760 | 14.8 | 26.1 | 2,152 | 21.6 | 73.9 | 2,912 | 19.3 | 100 |
| 35 - 44 | 637 | 12.4 | 32.7 | 1,313 | 13.2 | 67.3 | 1,950 | 12.9 | 100 |
| 45 - 54 | 934 | 18.1 | 36.7 | 1,610 | 16.2 | 63.3 | 2,544 | 16.8 | 100 |
| 55 - 64 | 1,022 | 19.8 | 38 | 1,665 | 16.7 | 62 | 2,687 | 17.8 | 100 |
| 65 - 74 | 877 | 17 | 40.2 | 1,305 | 13.1 | 59.8 | 2,182 | 14.4 | 100 |
| 75 - 79 | 324 | 6.3 | 36.4 | 566 | 5.7 | 63.6 | 890 | 5.9 | 100 |
| Total | 5,149 | 100 | 34.1 | 9,957 | 100 | 65.9 | 15,106 | 100 | 100 |
| **Sex** |  |  |  |  |  |  |  |  |  |
| Male | 2,482 | 48.2 | 32.7 | 5,110 | 51.3 | 67.3 | 7,592 | 50.3 | 100 |
| Female | 2,667 | 51.8 | 35.5 | 4,847 | 48.7 | 64.5 | 7,514 | 49.7 | 100 |
| Total | 5,149 | 100 | 34.1 | 9,957 | 100 | 65.9 | 15,106 | 100 | 100 |
| **Nationality** |  |  |  |  |  |  |  |  |  |
| Swiss | 4,269 | 82.9 | 37.4 | 7,147 | 71.8 | 62.6 | 11,416 | 75.6 | 100 |
| Non-Swiss | 880 | 17.1 | 23.8 | 2,810 | 28.2 | 76.2 | 3,690 | 24.4 | 100 |
| Total | 5,149 | 100 | 34.1 | 9,957 | 100 | 65.9 | 15,106 | 100 | 100 |
| **Number of household members** |  |  |  |  |  |  |  |  |  |
| 1 | 770 | 15 | 28.4 | 1,937 | 19.5 | 71.6 | 2,707 | 17.9 | 100 |
| 2 | 1,887 | 36.6 | 36.2 | 3,325 | 33.4 | 63.8 | 5,212 | 34.5 | 100 |
| 3-5 | 2,365 | 45.9 | 35.2 | 4,351 | 43.7 | 64.8 | 6,716 | 44.5 | 100 |
| 6 persons and more | 127 | 2.5 | 27 | 344 | 3.5 | 73 | 471 | 3.1 | 100 |
| Total | 5,149 | 100 | 34.1 | 9,957 | 100 | 65.9 | 15,106 | 100 | 100 |
| **Marital status** |  |  |  |  |  |  |  |  |  |
| single | 1,679 | 32.6 | 29.3 | 4,050 | 40.7 | 70.7 | 5,729 | 37.9 | 100 |
| married | 2,774 | 53.9 | 37.9 | 4,542 | 45.6 | 62.1 | 7,316 | 48.4 | 100 |
| widowed | 154 | 3 | 31.7 | 332 | 3.3 | 68.3 | 486 | 3.2 | 100 |
| divorced | 542 | 10.5 | 34.4 | 1,033 | 10.4 | 65.6 | 1,575 | 10.4 | 100 |
| Total | 5,149 | 100 | 34.1 | 9,957 | 100 | 65.9 | 15,106 | 100 | 100 |
| **Language region** |  |  |  |  |  |  |  |  |  |
| German | 2,282 | 44.3 | 32.8 | 4,675 | 47 | 67.2 | 6,957 | 46.1 | 100 |
| French | 1,387 | 26.9 | 34.2 | 2,674 | 26.9 | 65.8 | 4,061 | 26.9 | 100 |
| Italian | 1,480 | 28.7 | 36.2 | 2,608 | 26.2 | 63.8 | 4,088 | 27.1 | 100 |
| Total | 5,149 | 100 | 34.1 | 9,957 | 100 | 65.9 | 15,106 | 100 | 100 |
| **Urban/rural municipality** |  |  |  |  |  |  |  |  |  |
| urban | 3,139 | 61 | 32.8 | 6,418 | 64.5 | 67.2 | 9,557 | 63.3 | 100 |
| intermediary | 1,105 | 21.5 | 35.8 | 1,983 | 19.9 | 64.2 | 3,088 | 20.4 | 100 |
| rural | 905 | 17.6 | 36.8 | 1,556 | 15.6 | 63.2 | 2,461 | 16.3 | 100 |
| **Total** | **5,149** | **100** | **34.1** | **9,957** | **100** | **65.9** | **15,106** | **100** | **100** |
