# Supplementary material for: Public willingness to participate in personalized health research and biobanking: A large-scale Swiss survey
Source: PLoS One. 2021 Apr 1;16(4):e0249141. doi: 10.1371/journal.pone.0249141 (PMC8016315; doi:10.1371/journal.pone.0249141)
Supplement: S2 Table — Not weighted. (DOCX) [file pone.0249141.s002.docx]

**S2 Table. Socio-demographic characteristics displayed for chosen type of response (not weighted).**

|  | | **Type of response** | | | |
| --- | --- | --- | --- | --- | --- |
|  | **Web-based** | | | **Paper-based** | |
|  | | **N** | **%** | **N** | **%** |
| **Age group** | |  |  |  |  |
| 18 - 24 | | 463 | 13.2 | 131 | 8.4 |
| 25 - 34 | | 551 | 15.7 | 207 | 13.2 |
| 35 - 44 | | 469 | 13.3 | 163 | 10.4 |
| 45 - 54 | | 675 | 19.2 | 252 | 16.1 |
| 55 - 64 | | 691 | 19.6 | 314 | 20 |
| 65 - 74 | | 513 | 14.6 | 344 | 22 |
| 75 - 79 | | 157 | 4.5 | 156 | 10 |
| Total | | 3519 | 100 | 1567 | 100 |
| **Sex** | |  |  |  |  |
| Male | | 1757 | 49.9 | 694 | 44.3 |
| Female | | 1762 | 50.1 | 873 | 55.7 |
| Total | | 3519 | 100 | 1567 | 100 |
| **Nationality** | |  |  |  |  |
| Swiss | | 2946 | 83.7 | 1270 | 81 |
| Non-Swiss | | 573 | 16.3 | 297 | 19 |
| Total | | 3519 | 100 | 1567 | 100 |
| **Number of household members** | |  |  |  |  |
| 1 | | 483 | 13.7 | 277 | 17.7 |
| 2 | | 1199 | 34.1 | 655 | 41.8 |
| 3-5 | | 1748 | 49.7 | 599 | 38.2 |
| 6 persons and more | | 89 | 2.5 | 36 | 2.3 |
| Total | | 3519 | 100 | 1567 | 100 |
| **Marital status** | |  |  |  |  |
| Single | | 1237 | 35.2 | 432 | 27.6 |
| Married | | 1862 | 52.9 | 871 | 55.6 |
| Widowed | | 72 | 2 | 78 | 5 |
| Divorced | | 348 | 9.9 | 186 | 11.9 |
| Total | | 3519 | 100 | 1567 | 100 |
| **Biological children** | |  |  |  |  |
| Yes | | 1993 | 56.6 | 975 | 62.2 |
| No | | 1526 | 43.4 | 548 | 35 |
| Missing | | 0 | 0 | 44 | 2.8 |
| Total | | 3519 | 100 | 1567 | 100 |
| **Language region** | |  |  |  |  |
| German | | 1613 | 45.8 | 644 | 41.1 |
| French | | 933 | 26.5 | 433 | 27.6 |
| Italian | | 973 | 27.6 | 490 | 31.3 |
| Total | | 3519 | 100 | 1567 | 100 |
| **Urban/rural municipality** | |  |  |  |  |
| Urban | | 2178 | 61.9 | 926 | 59.1 |
| Intermediary | | 750 | 21.3 | 336 | 21.4 |
| Rural | | 591 | 16.8 | 305 | 19.5 |
| Total | | 3519 | 100 | 1567 | 100 |
| **Education** | |  |  |  |  |
| Compulsory education or less | | 205 | 5.8 | 180 | 11.5 |
| Upper secondary education | | 2331 | 66.2 | 1063 | 67.8 |
| Tertiary education | | 983 | 27.9 | 300 | 19.1 |
| Missing | | 0 | 0 | 24 | 1.5 |
| Total | | 3519 | 100 | 1567 | 100 |
| **Religion** | |  |  |  |  |
| Very much | | 431 | 12.2 | 264 | 16.8 |
| Somewhat | | 1531 | 43.5 | 686 | 43.8 |
| Not at all | | 1557 | 44.2 | 581 | 37.1 |
| Missing | | 0 | 0 | 36 | 2.3 |
| Total | | 3519 | 100 | 1567 | 100 |
| **Working in health sector?** | |  |  |  |  |
| Yes | | 697 | 19.8 | 313 | 20 |
| No | | 2822 | 80.2 | 1241 | 79.2 |
| Missing | | 0 | 0 | 13 | 0.8 |
| Total | | 3519 | 100 | 1567 | 100 |
| **Health status** | |  |  |  |  |
| Very unhealthy | | 40 | 1.1 | 21 | 1.3 |
| Somewhat unhealthy | | 58 | 1.6 | 57 | 3.6 |
| Neutral | | 393 | 11.2 | 268 | 17.1 |
| Somewhat healthy | | 1753 | 49.8 | 825 | 52.6 |
| Very healthy | | 1275 | 36.2 | 368 | 23.5 |
| Missing | | 0 | 0 | 28 | 1.8 |
| Total | | 3519 | 100 | 1567 | 100 |
