# Supplementary material for: Public willingness to participate in personalized health research and biobanking: A large-scale Swiss survey
Source: PLoS One. 2021 Apr 1;16(4):e0249141. doi: 10.1371/journal.pone.0249141 (PMC8016315; doi:10.1371/journal.pone.0249141)
Supplement: S1 File — (DOCX) [file pone.0249141.s003.docx]

**S1 File**

Question 2: What is your general opinion towards research with human biological samples (like blood, saliva, urine, hair or tissue)?

| Category | No. | Percent |
| --- | --- | --- |
| Very negative | 53 | 1 |
| Somewhat negative | 209 | 4.1 |
| Neutral | 1,355 | 26.8 |
| Somewhat positive | 2,424 | 47.9 |
| Very positive | 1,021 | 20.2 |
| Total | 5,062 | 100 |

Question 4: Would you participate in a research project that used your health data (disease history and medical records) and/or biological samples (like blood, saliva, urine, hair or tissue)?

| Category | No. | Percent |
| --- | --- | --- |
| Yes | 2,726 | 54.1 |
| No | 2,317 | 45.9 |
| Total | 5,043 | 100 |

Question 4.1: If yes, which types of data and samples would you hypothetically be willing to provide? *Please select as many as apply.*

| Possible answers | No. |
| --- | --- |
| Questionnaires about my health status | 2,352 |
| Medical files about myself | 1,642 |
| My family's medical history | 1,382 |
| My blood sample | 2,326 |
| Biological samples I can take myself | 2,224 |
| Biological samples taken by medical staff | 1,658 |
| Social Media data | 392 |
| Derived from apps about my health or lifestyle | 994 |

Question 5: Which of the below concerns would you have? *Please select up to 3 most relevant ones.*

| Possible answers | No. |
| --- | --- |
| Worried about research involving information about my genes | 765 |
| Afraid of what I might discover about my own health risks | 670 |
| Worried my data won't be kept confidential | 2,328 |
| Worried someone may hack and steal my data | 1,601 |
| Worried data used to discriminate against me or my family | 2,354 |
| Worried data misused for commercial or marketing purposes | 2,286 |
| I don't want other to benefit financially from my data | 1,570 |
| I am afraid of needles or the procedures to get the samples | 321 |
| I don't want to make the effort required to donate | 395 |
| I don't receive money for it | 149 |
| I don't have time to contribute | 370 |
| I do not care about health research | 61 |
| No specific reason | 467 |
| Other (please specify): | 34 |

Question 12: Imagine you participated in a biobank’s study and it were possible to receive personal research results: Which types of research results would you like to receive?

| Sub-question | Yes | No | Don't know | Total |
| --- | --- | --- | --- | --- |
| Basic medical information | 4,083 | 471 | 409 | 4,963 |
|  | 82.3% | 9.5% | 8.2% | 100% |
| Lifestyle affects my risk of getting a medical condition | 3,488 | 1,031 | 410 | 4,929 |
|  | 70.8% | 20.9% | 8.3% | 100% |
| Diseases for which medical treatments are available | 3,732 | 715 | 509 | 4,956 |
|  | 75.3% | 14.4% | 10.3% | 100% |
| Diseases for which only preventive actions can be undertaken | 3,687 | 764 | 480 | 4,931 |
|  | 74.8% | 15.5% | 9.7% | 100% |
| Diseases for which no treatment is available | 2,809 | 1,375 | 739 | 4,923 |
|  | 57.1% | 27.9% | 15.0% | 100% |
| Research results about the study in general | 3,282 | 926 | 734 | 4,942 |
|  | 66.4% | 18.7% | 14.9% | 100% |
