# Supplementary material for: Public willingness to participate in personalized health research and biobanking: A large-scale Swiss survey
Source: PLoS One. 2021 Apr 1;16(4):e0249141. doi: 10.1371/journal.pone.0249141 (PMC8016315; doi:10.1371/journal.pone.0249141)
Supplement: S2 File — (PDF) [file pone.0249141.s004.pdf]

ETH
Eidgenössische Technische Hochschule Zürich
Swiss Federal Institute of Technology Zurich

UNIVERSITÄT BERN

Health Ethics & Policy Lab
Chair of Bioethics, ETH Zurich

Institutr for Social and Preventive Medicine
University of Bern

Tel: 044 505 15 13
E-mail: persmed@ethz.ch

Swiss Survey

Your views on personalised health research

a joint survey by ETH Zurich, Health Ethics & Policy Lab and University Bern, Institute for Social and Preventive Medicine

Online Survey:

www.persmed.ethz.ch

Password: *XYZ*

Introduction

Welcome to our survey on personalised health research!

Imagine you are invited to by a Swiss publicly funded personalised health research project. That hypothetical project collects data about your health.

The types of data collected are personal data (gender, age, weight etc.), health data (disease history and medical records), and biological samples (blood, saliva, urine, hair or tissue), which can give information about your DNA. In the following we will refer to them as data and samples.

These data and samples would be stored in a Swiss biobank. This is a large databank run by public research centres like universities. The Data and samples are kept confidential and secure.

With this survey we want to know more about your attitude towards personalised health research.

In particular, we are interested in whether and under which circumstances you would be willing to provide your data and/or samples to a Swiss biobank. What concerns would you have? What would be your expectations? To get a better understanding of what is important to you, please answer the following questions.

The survey does not aim to test your knowledge about this topic, but rather to get your opinions and views. There are no right and wrong answers. It is only important that you answer the questions truthfully and to the best to your abilities.

Thank you very much for taking the time to fill out this survey!

Extra information on personalised health research:

Why do people with the same illnesses react so differently to treatments? Why does a drug work very well in one person and not in another? And why does one person develop a disease and others do not?

Research in **personalised health wants to** answer these questions in order to **treat sick people in a more precise, personalised and effective manner** in the future. Personalised health research also uses genetic and other personal information of healthy individuals to learn more about the causes of certain diseases and chances of developing those.

For doing so, it is important that researchers have access to as many available health data and samples of different persons (with different health status) as possible in the biobanks that are made.

How should I fill out the questionnaire?

- Tick the applicable box next to the selected answer: ☒
- If you accidentally check the wrong box, fill it in completely with a ballpoint pen and then check the applicable box.

Part 1: Your attitude

We would like to begin with your general perspective on the following issues:

Q1. Before today, have you ever heard anything about research with biological samples (e.g. blood, saliva, urine, hair or tissue)?

☐ Yes ☐ No

Q2. What is your general opinion towards research with human biological samples (like blood, saliva, urine, hair or tissue)?

☐	☐	☐	☐	☐
Very Negative	Somewhat negative	Indifferent	Somewhat positive	Very Positive

Q3. In your opinion, how valuable do you think it is for you, your family, or society if you participate in research by providing your health data (disease history and medical records) and biological samples (like blood, saliva, urine, hair or tissue)?

Please rate on a scale from 1 = no value to 5 = high value.

	1	2	3	4	5	Don’t know
1. For me personally	☐	☐	☐	☐	☐	☐
2. For my family	☐	☐	☐	☐	☐	☐
3. For society	☐	☐	☐	☐	☐	☐

Q4. Would you participate in a research project that used your health data (disease history and medical records) and/or biological samples (like blood, saliva, urine, hair or tissue)?

☐ Yes (go on with question **4.1**)

☐ No (go on with question **5**)

Note: Even if you answered no to this question, we would like to ask you to fully complete this survey. We are interested in your opinion how research with health data and biological samples should best be set up according to your view.

Q4.1 If **yes**, which types of data and samples would you hypothetically be willing to provide?

Please select as many as apply.

☐ Questionnaires about my health status

☐ Medical files about myself (digital or on paper)

☐ My family's medical history

☐ My blood sample

☐ My biological samples that I can take myself (e.g. saliva, hair, urine, buccal swab)

☐ My biological samples that have to be taken by medical staff (e.g. tumor tissue or skin cells)

☐ My Social Media data

☐ Data derived from apps about my health or lifestyle, e.g. exercise tracker, food log, heart rate, etc.

Q4.2 If **yes**, why you would participate in this research?

Please select up to 3 reasons which are most relevant to you.

☐ To increase scientific knowledge

☐ To benefit society and future generations

☐ General sense of duty

☐ To improve overall healthcare

☐ To gain personal benefit by learning something about my health

☐ To benefit my family (to know about genetically inherited diseases)

☐ Other reason (please specify): ______________________________

☐ I don't know

Q5. Which of the below concerns would you have?

Please select up to 3 most relevant ones.

☐ I am worried about research involving information about my genes

☐ I am afraid of what I might discover about my own health risks

☐ I am worried my data won't be kept confidential

☐ I am worried someone may hack and steal my data

☐ I am worried someone may use my data to discriminate against me or my family (e.g. by health insurances or financial services)

☐ I am worried that my data and samples may be misused for commercial or marketing purposes instead of research

☐ I don’t want others (like private companies or researchers) to benefit financially from my data

☐ I am afraid of needles or the procedures to get the samples

☐ I don’t want to make the effort required to donate

☐ I don’t receive money for it

☐ I don’t have time to contribute

☐ I do not care about health research

☐ No specific reason

☐ Other (please specify):

____

____

Q6. Whether you would donate your data and samples to a hypothetical biobank or not: What information is most important to you in order to make the decision?

Please select the 3 types of information most important to you:

☐ The exact types of research which will be conducted

☐ The potential benefits and risks of donating my data and samples

☐ The way data and samples are stored, such as made anonymous, encoded or stored together with my name

☐ Who has access to my data and samples

☐ Who will benefit from the research

☐ Who will financially profit from the research

☐ The security measures to keep data and samples private and protected

☐ None of this information would help me decide

☐ Other (please specify):

____

____

Part 2: Data management

In this part, we would like to know how you think the provided data and samples should be managed.

Q7. Biobanks would like to use your data and samples in multiple research projects, also in the future. How often would you like to be asked by the biobank for permission to use your data and samples in research projects? (Your decision could always be changed or withdrawn.)

☐	☐	☐	☐
Ask me only once, when I donate my data and/or samples	Ask me again for every new project	It would depend on the type of project that is being considered	Not sure

Q8. In which form would you prefer your data and samples to be stored?

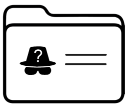

☐ **Anonymized**

Explanation:
Data & samples that are anonymized cannot possibly be linked back to a specific person without enormous effort.
This means identifiers – such as your name, address, pictures – are removed from your data.

Consequences:
→ You would only receive general research results about the research project.

Privacy risk: Low
(There are limited risks to your privacy, because it is very difficult to link your data back to you.)

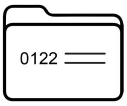

☐ **Coded**

Explanation*:*
Data & samples are linked to a specific person via a code that is securely stored by researchers.
This means a code/key is required to link data back to you.

Consequences:
→ You receive individual results about your personal status in addition to general research results about the research project.

Privacy risk*:* Medium
(There are limited risks to your privacy, because your data can only be identified with a secure code.)

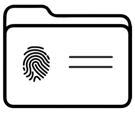

☐ **Identifiable**

Erklärung:
Data & samples are stored together and labelled with your name. They can easily be linked back to you.

Consequences:
→ You receive individual results about your personal status in addition to general research results about the research project.

Privacy risk: High
(Your privacy can be at risk, because it is easy to identify your data.)

Q9. Who do you think should own the data and samples you donate to a biobank?

Please select one answer.

☐ Me personally

☐ The biobank

☐ The Swiss government

☐ The universities involved with the biobank

☐ The specific researchers who make discoveries

☐ No one

☐ Other (please specify): ____

☐ I am not sure

Part 3: Data governance and sharing

Now, we would like to know how you think data and samples should be governed and shared.

Q10. In your opinion, who should be responsible for ensuring that your data and samples are correctly stored and managed?

Please select one answer.

☐ The biobank (management board)

☐ An independent committee representing the public (e.g. citizens, patients, the public)

☐ An independent expert committee (e.g. independent researchers: scientists and clinicians not associated with the biobank)

☐ A mixed committee of the public and experts

☐ Other (please specify): ____

Q11. Imagine the following people and organisations in Switzerland have access to your data and samples in the biobank. To what extent would you trust them to keep your data and samples confidential and protected?

Please rate on a scale from 1 = no trust to 5 = strong trust.

	1	2	3	4	5
My doctor	☐	☐	☐	☐	☐
Doctors in general	☐	☐	☐	☐	☐
Researchers at a university	☐	☐	☐	☐	☐
Researchers at other public institutes	☐	☐	☐	☐	☐
Pharma companies	☐	☐	☐	☐	☐
Other global private, for-profit companies	☐	☐	☐	☐	☐
Other Swiss private, for-profit companies	☐	☐	☐	☐	☐
Health insurances	☐	☐	☐	☐	☐
Swiss government	☐	☐	☐	☐	☐

Part 4: Your research results

In this part, we would like to know what research results you would like to receive if you donated data and samples to a study.

Q12. Imagine you participated in a biobank's study and it were possible to receive personal research results: Which types of research results would you like to receive?

	Yes	No	Don't know
1. Basic medical information, e.g. lab results such as blood count	☐	☐	☐
2. How my lifestyle (e.g. smoking, weight etc.) affects my risk of getting a medical condition	☐	☐	☐
3. Results about risks of diseases for which medical treatments or interventions are available (e.g. risk for some types of cancer, some heart problems)	☐	☐	☐
4. Results about risks of diseases for which only preventive actions can be undertaken (e.g. risk for diabetes or heart diseases)	☐	☐	☐
5. Results about risks of diseases for which no medical treatment is available but they could impact my well-being or decisions about my career or family planning, (e.g. Alzheimer's disease/dementia)	☐	☐	☐
6. Research results about the study in general (the results do not apply to me specifically)	☐	☐	☐

Q13. Sometimes genetic results of developing a disease can only be expressed in a likelihood of developing the disease. Which level of likelihood for developing a disease would you want to get?

I want to receive my results…

☐ ... when I will **certainly** get the disease.

☐ ... when it is **highly likely** that I get the disease.

☐ ... when it is possible, but **not very likely** that I get the disease.

☐ I don't want to receive any results about likelihoods of diseases.

☐ I don't know.

Q14. If you want to receive results, through which communication channel would you like to receive them?

Please select all that apply.

☐ Letter in the mail

☐ Phone call

☐ EMail

☐ Website with secure login

☐ Mobile App

☐ Personal communication (face-to-face)

Q15. How important would money or any other material compensation be for you to provide your data and samples to a Swiss publicly funded biobank?

Please rate on a scale from 1 = not important to 5 = very important.

1	2	3	4	5
☐	☐	☐	☐	☐

Finally: Personal information

In this final part, we ask you to provide some information about yourself.

Q16. How would you describe your overall health status?

☐	☐	☐	☐	☐
Very unhealthy	Somewhat unhealthy	Neutral	Somewhat healthy	Very healthy

Q17. How old are you?

☐ 18-24 ☐ 35-44 ☐ 55-64 ☐ 75 or older

☐ 25-34 ☐ 45-54 ☐ 65-74

Q18. What is your gender?

☐ Male ☐ Other

☐ Female ☐ Prefer not to disclose

Q19. Do you have biological children?

☐ Yes ☐ No

Q20. What is your nationality?
Please select all that apply.

☐ Swiss ☐ German ☐ Kosovo

☐ Italian ☐ Portuguese ☐ Other/additional (pease specify): ____________________

☐ French ☐ Spanish

Q21. What is your highest completed level of education?

☐ None or up to 7 years obligatory school

☐ Obligatory school (8 or 9 years)

☐ Basic vocational training / apprenticeship

☐ High School (Matura)

☐ Higher vocational training / apprenticeship or technical school

☐ University degree (Bachelor / Master / Doctoral degree)

Q22. Do you currently work or have ever worked in the health care or health research sector?

☐ Yes ☐ No

Q23. Would you describe yourself as a religious person?

☐	☐	☐
Very much	Somewhat	Not at all

General comments:

End of the survey

Thank you very much for taking the time to fill out this survey!
We highly appreciate your valuable contribution!

Please send back the completed questionnaire until 23rd of November 2019 to the following address. You can use the enclosed pre-stamped envelope for this.

Health Ethics and Policy Lab
ETH Zürich
Hottingerstrasse 10
8092 Zürich
