# Supplementary material for: Public willingness to participate in personalized health research and biobanking: A large-scale Swiss survey
Source: PLoS One. 2021 Apr 1;16(4):e0249141. doi: 10.1371/journal.pone.0249141 (PMC8016315; doi:10.1371/journal.pone.0249141)
Supplement: S3 File — (PDF) [file pone.0249141.s005.pdf]

ETH
Eidgenössische Technische Hochschule Zürich
Swiss Federal Institute of Technology Zurich

UNIVERSITÄT BERN

Health Ethics & Policy Lab
Lehrstuhl für Bioethik, ETH Zürich

Institut für Sozial- und Präventivmedizin
Universität Bern

Tel: 044 505 15 13
E-mail: persmed@ethz.ch

Schweizer Umfrage

Ihre Meinung zu personalisierter Gesundheitsforschung

eine gemeinsame Umfrage von ETH Zürich, Health Ethics & Policy Lab und Universität Bern, Institut für Sozial- und Präventivmedizin

Online-Umfrage:

www.persmed.ethz.ch

Passwort: XYZ

Einführung

Willkommen zu unserer Umfrage zu personalisierter Gesundheitsforschung!

Stellen Sie sich vor, Sie werden gefragt, Ihre Daten einem Schweizer Forschungsprojekt zu personalisierter Gesundheit zur Verfügung zu stellen. Das hypothetische Projekt sammelt Daten über Ihre Gesundheit.

Bei den gesammelten Daten würde es sich um personenbezogene Daten (Geschlecht, Alter, Gewicht usw.), gesundheitsbezogene Daten (Krankheitsgeschichte und medizinische Informationen) und biologische Proben (Blut, Speichel, Urin, Haare oder Gewebe) handeln, die Auskunft über Ihre DNA geben können.

Im Folgenden werden diese gesammelten Daten nur als Daten und Proben bezeichnet. Diese Daten und Proben würden in einer Schweizer Biobank gespeichert. Dies ist eine grosse Datenbank, die von öffentlichen Forschungszentren wie Universitäten betrieben wird. Die Daten werden vertraulich und sicher behandelt.

Mit dieser Umfrage wollen wir mehr über Ihre Einstellungen zu personalisierter Gesundheitsforschung erfahren.

Insbesondere interessiert uns, ob und unter welchen Umständen, Sie dazu bereit wären, Ihre Daten und/oder Proben einer Schweizer Biobank zur Verfügung zu stellen. Welche Bedenken hätten Sie? Was wären Ihre Erwartungen? Um ein besseres Verständnis dafür zu bekommen, was Ihnen wichtig ist, beantworten Sie bitte die folgenden Fragen.

Die Umfrage zielt nicht darauf ab Ihr Wissen zu testen sondern möchte vielmehr Ihre Meinungen und Ansichten über die Bereitstellung von Daten und Proben für die Forschung erfahren. Es gibt keine richtigen und falschen Antworten. Wichtig ist nur, dass Sie die Fragen ehrlich beantworten.

Vielen Dank, dass Sie sich die Zeit nehmen, diese Umfrage auszufüllen!

Zusätzliche Informationen zu personalisierter Gesundheitsforschung:

Warum reagieren Menschen mit den gleichen Krankheiten so unterschiedlich auf Behandlungen? Warum wirkt ein Medikament bei einem Menschen sehr gut und bei einem anderen nicht? Und warum entwickelt der eine Mensch eine Krankheit und der andere nicht?

Die **Forschung im Bereich der personalisierten Gesundheit will** diese Fragen beantworten, um **erkrankte Menschen in Zukunft präziser, personalisierter und effektiver zu behandeln.** Die personalisierte Gesundheitsforschung nutzt auch genetische und weitere Informationen von gesunden Menschen, um mehr über die Ursachen bestimmter Krankheiten und die Wahrscheinlichkeiten für deren Entstehung zu erfahren. Dabei ist es wichtig, dass Forscher auf möglichst viele verfügbare Gesundheitsdaten verschiedener Personen zugreifen können.

Wie muss ich den Fragebogen ausfüllen?

- Kreuzen Sie das zutreffende Kästchen neben der gewählten Antwort an: ☒
- Wenn Sie versehentlich das falsche Kästchen angekreuzt haben, so füllen Sie dieses mit dem Kugelschreiber ganz aus und kreuzen Sie dann das zutreffende Kästchen an.

Teil 1: Ihre Einstellung

Zuerst möchten wir gerne Ihre allgemeine Einstellung zu den folgenden Themen erfahren:

Q1. Haben Sie vor dieser Umfrage schon einmal etwas über Forschung mit biologischen Proben (z.B. Blut, Speichel, Urin, Haare oder Gewebe) gehört?

☐ Ja ☐ Nein

Q2. Wie ist Ihre Meinung zur Forschung mit biologischen menschlichen Proben (wie Blut, Speichel, Urin, Haare oder Gewebe)?

☐	☐	☐	☐	☐
Sehr negativ	Eher negativ	Neutral	Eher positiv	Sehr positiv

Q3. Wie gross bewerten Sie den Nutzen für Sie persönlich, Ihre Familie und die Gesellschaft, wenn Sie Ihre Daten und/oder Proben der Forschung zur Verfügung stellen würden?

Bitte bewerten Sie auf einer Skala von 1 = kein Nutzen bis 5 = grosser Nutzen.

	1	2	3	4	5	Weiss nicht
1. Für mich persönlich	☐	☐	☐	☐	☐	☐
2. Für meine Familie	☐	☐	☐	☐	☐	☐
3. Für die Gesellschaft allgemein	☐	☐	☐	☐	☐	☐

Q4. Würden Sie an einem Forschungsprojekt teilnehmen, das Ihre Daten und/oder Proben verwendet?

☐ Ja (weiter mit Frage **4.1**)

☐ Nein (weiter mit Frage **5**)

Hinweis: Auch wenn Sie diese Frage mit Nein beantwortet haben, möchten wir Sie bitten, diese Umfrage vollständig auszufüllen. Wir sind an Ihrer Meinung interessiert, wie Forschung mit Daten und Proben Ihren Vorstellungen nach am besten gestaltet werden sollte.

Q4.1 Wenn **ja**, welche Art von Daten wären Sie hypothetisch bereit zur Verfügung zu stellen?

Kreuzen Sie bitte alle zutreffenden Antworten an.

☐ Fragebögen über meinen Gesundheitszustand

☐ Medizinische Unterlagen über mich (digital oder als Akte)

☐ Krankengeschichte meiner Familie

☐ Blutprobe

☐ Meine biologischen Proben, die ich selbst entnehmen kann (z.B. Speichel, Haare, Urin, Wangenabstrich)

☐ Meine biologische Proben, die von medizinischem Personal entnommen werden (z.B. Tumorgewebe, Hautzellen)

☐ Daten aus sozialen Medien

☐ Daten, die von Apps über meine Gesundheit oder meinen Lebensstil gesammelt werden, z.B. Fitness-Tracker, Ernährungstagebuch, Herzfrequenz, etc.

Q4.2 Wenn **ja**, warum würden Sie an dieser Forschung teilnehmen?

Kreuzen Sie bitte bis zu 3 für Sie relevante Gründe an.

☐ Um wissenschaftliche Erkenntnisse voranzutreiben

☐ Nutzen für die Gesellschaft und zukünftige Generationen

☐ Allgemeines Pflichtbewusstsein

☐ Verbesserung der allgemeinen Gesundheitsversorgung

☐ Aussicht aus der Forschung einen persönlichen Nutzen zu ziehen, indem ich etwas über meine Gesundheit erfahre

☐ Aussicht mit der Forschung meiner Familie zu helfen (um z.B. etwas über genetisch vererbte Krankheiten zu erfahren)

☐ Sonstige Gründe (bitte angeben): ____

☐ Weiss nicht

Q5. Welche der folgenden Bedenken haben Sie gegenüber Biobankforschung?

Kreuzen Sie bitte bis zu 3 für Sie relevante Gründe an.

☐ Ich habe Bedenken gegenüber Forschung, die meine genetischen Informationen verwendet

☐ Ich habe Angst davor, etwas über meine eigenen Gesundheitsrisiken zu erfahren

☐ Ich habe Angst, dass meine Daten nicht vertraulich behandelt werden

☐ Ich bin besorgt, dass jemand meine Daten hacken und stehlen könnte

☐ Ich habe Angst, dass jemand meine Daten verwendet, um mich oder meine Familie zu diskriminieren (z.B. Krankenkassen oder Banken)

☐ Ich bin besorgt, dass meine Daten und/oder Proben für kommerzielle oder Marketingzwecke verwendet werden könnten

☐ Ich möchte nicht, dass andere (wie private Unternehmen oder Forscher) finanziell von meinen Daten profitieren

☐ Ich habe Angst vor Nadeln oder den Verfahren, um Proben zu entnehmen

☐ Ich möchte den erforderlichen Aufwand nicht betreiben

☐ Ich bekomme kein Geld dafür

☐ Ich habe keine Zeit

☐ Ich interessiere mich nicht für Gesundheitsforschung

☐ Kein besonderer Grund

☐ Sonstige Gründe (bitte angeben):

____

____

Q6. Unabhängig davon, ob Sie Ihre Daten und/oder Proben einer Biobank zur Verfügung stellen würden oder nicht: Welche Informationen würden Sie gerne vorab erhalten?

Kreuzen Sie bitte bis zu 3 für Sie wichtigsten Arten von Informationen an.

☐ Die genaue Art der Forschung, die durchgeführt werden soll

☐ Die potenziellen Nutzen und Risiken wenn ich meine Daten und/oder Proben zur Verfügung stelle

☐ Die Art und Weise wie Daten und Proben gespeichert werden, z.B. anonymisiert, verschlüsselt oder mit meinem Namen

☐ Wer Zugriff auf meine Daten und Proben hat

☐ Wem genau die Forschung nutzen wird

☐ Wer finanziell von der Forschung profitiert

☐ Die Sicherheitsmaßnahmen zur Wahrung von Privatsphäre und Datenschutz

☐ Keine dieser Informationen würde mir helfen eine Entscheidung zu treffen

☐ Sonstige (bitte angeben):

____

____

Teil 2: Datenmanagement

In diesem Teil möchten wir wissen, wie Daten und Proben Ihrer Meinung nach von Biobanken gespeichert und verwaltet werden sollten.

Q7. Biobanken möchten Ihre Daten und Proben auch in zukünftigen Forschungsprojekten verwenden. Wie oft möchten Sie von der Biobank um Erlaubnis gebeten werden, damit Ihre Daten und/oder Proben in zukünftigen Forschungsprojekten verwendet werden können? (Sie könnten Ihre Entscheidung jederzeit ändern oder widerrufen.)

- ☐ Nur einmal, wenn ich meine Daten und/oder Proben abgebe
- ☐ Erneut zu jedem neuen Projekt
- ☐ Es hängt von der Art des Projekts ab, das in Betracht gezogen wird
- ☐ Weiss nicht

Q8. In welcher Form möchten Sie, dass Ihre Daten und/oder Proben gespeichert werden?

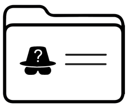

☐ **Anonymisiert**

Erklärung:

Daten & Proben sind anonymisiert und können ohne enormen Aufwand nicht auf eine bestimmte Person zurückgeführt werden. Das bedeutet, dass Identifikatoren - wie Ihr Name, Adresse, Bilder - aus Ihren Daten entfernt werden.

Konsequenzen:

→ Sie könnten nur allgemeine Forschungsergebnisse über das gesamte Forschungsprojekt erhalten.

Datenschutzrisiko: Niedrig

(Die Risiken für Ihre Privatsphäre sind gering, da es sehr schwierig und nur mit grossem Aufwand möglich ist, Ihre Daten mit Ihnen zu verbinden.)

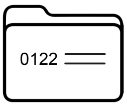

☐ **Verschlüsselt**

Erklärung:

Daten & Proben werden mit einem Code versehen, der von Forschern sicher abgespeichert wird. Nur mit diesem Code kann ein Bezug zwischen Ihren Daten und Ihnen hergestellt werden.

Konsequenzen:

→ Sie könnten allgemeine Forschungsergebnisse über das gesamte Projekt sowie indivi-duelle Ergebnisse über Ihren persönlichen Gesundheitsstatus erhalten.

Datenschutzrisiko: Mittel

(Die Risiken für Ihre Privatsphäre sind begrenzt, da Ihre Daten nur mit einem sicheren Code identifiziert werden können.)

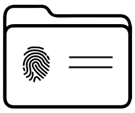

☐ **Identifizierbar**

Erklärung:

Daten & Proben werden zusammen mit Ihrem Namen abgespeichert und können leicht auf Sie zurückgeführt werden.

Konsequenzen:

→ Sie könnten allgemeine Forschungsergebnisse über das gesamte Projekt sowie indivi-duelle Ergebnisse über Ihren persönlichen Gesundheitsstatus erhalten

Datenschutzrisiko: Hoch

(Ihre Privatsphäre kann gefährdet sein, da es leicht ist, Ihre Daten mit Ihnen zu verbinden.)

Q9. Wem sollten Ihrer Meinung nach die Daten und Proben gehören, die Sie einer Biobank zur Verfügung stellen?

Bitte wählen Sie eine Antwort aus.

☐ Mir persönlich

☐ Der Biobank

☐ Dem Schweizer Staat

☐ Den Universitäten, die an der Biobank beteiligt sind

☐ Den spezifischen Forschern, die wissenschaftliche Entdeckungen machen

☐ Niemandem

☐ Sonstige (bitte angeben): ____

☐ Weiss nicht

Teil 3: Umgang mit Daten & deren Weitergabe

Nun möchten wir Ihre Meinung darüber erfahren, wie mit den zur Verfügung gestellten Daten umgegangen und an wen sie weitergegeben werden sollten.

Q10. Wer sollte Ihrer Meinung nach dafür verantwortlich sein sicherzustellen, dass Ihre Daten und Proben korrekt gespeichert und verwaltet werden?

Kreuzen Sie bitte eine Antwort an.

☐ Die Biobank (Geschäftsführung)

☐ Ein unabhängiger Ausschuss, der die Bevölkerung repräsentiert (z.B. Bürger, Patienten etc.)

☐ Ein unabhängiges Expertengremium (z.B. unabhängige Forscher: Wissenschaftler und Ärzte, die nicht mit der Biobank verbunden sind)

☐ Ein gemischter Ausschuss: Bürger und Experten

☐ Sonstige (bitte angeben): ______________________________

Q11. Stellen Sie sich vor, die folgenden Personen und Organisationen in der Schweiz haben Zugang zu Ihren in einer Biobank gelagerten Daten und Proben. Inwieweit würden Sie den folgenden Personen und Organisationen vertrauen, dass sie Ihre Daten und Proben vertraulich und geschützt behandeln?

Bitte bewerten Sie auf einer Skala von 1 = kein Vertrauen bis 5 = grosses Vertrauen.

	1	2	3	4	5
Mein Arzt	☐	☐	☐	☐	☐
Ärzte im Allgemeinen	☐	☐	☐	☐	☐
Forscher an einer Universität	☐	☐	☐	☐	☐
Forscher an anderen öffentlichen Einrichtungen	☐	☐	☐	☐	☐
Pharmaunternehmen	☐	☐	☐	☐	☐
Andere private & gewinnorientierte Unternehmen weltweit	☐	☐	☐	☐	☐
Andere private & gewinnorientierte Unternehmen in der Schweiz	☐	☐	☐	☐	☐
Krankenversicherungen	☐	☐	☐	☐	☐
Schweizer Staat	☐	☐	☐	☐	☐

Teil 4: Ihre Forschungsergebnisse

In diesem Teil möchten wir wissen, welche Forschungsergebnisse Sie gerne erhalten würden, wenn Sie Ihre Daten und/oder Proben für Biobankstudien zur Verfügung stellen würden.

Q12. Stellen Sie sich vor, Sie hätten an einem Forschungsprojekt teilgenommen und es wäre möglich individuelle Forschungsergebnisse zu erhalten: Welche Arten von Forschungsergebnissen würden Sie gerne erhalten?

	Ja	Nein	Weiss nicht
1. Medizinische Basisinformationen, wie z.B. Blutwerte	☐	☐	☐
2. Wie sich mein Lebensstil auf mein Risiko auswirkt, an einer Krankheit zu erkranken (z.B. Rauchen, Gewicht usw.)	☐	☐	☐
3. Ergebnisse über Krankheitsrisiken, für die medizinische Behandlungen zur Verfügung stehen (z.B. Risiko für bestimmte Krebsarten, Herzprobleme)	☐	☐	☐
4. Ergebnisse über Krankheitsrisiken, für die nur vorbeugende Massnahmen ergriffen werden können (z.B. Risiko für Diabetes oder Herz-Kreislauf-Erkrankungen)	☐	☐	☐
5. Ergebnisse über Krankheitsrisiken, für die es keine Heilung gibt, die aber meine Entscheidungen bzgl. Karriere oder Familienplanung beeinflussen können (z.B. Alzheimer/Demenz)	☐	☐	☐
6. Allgemeine Forschungsergebnisse zur Studie (keine persönlichen Ergebnisse, sondern nur Gesamt-ergebnisse)	☐	☐	☐

Q13. Manchmal können nur Wahrscheinlichkeiten angegeben werden, ob jemand eine genetische Krankheit entwickelt oder nicht. Welche Wahrscheinlichkeit für ein Krankheitsrisiko würden Sie erhalten wollen?

Ich möchte Ergebnisse zu meinen Krankheisrisiken erhalten...

☐ ... wenn ich eine Krankheit **mit Sicherheit** bekommen werde.

☐ ... wenn es **sehr wahrscheinlich** ist, dass ich eine Krankheit bekomme.

☐ ... wenn es möglich, aber **nicht sehr wahrscheinlich** ist, dass ich eine Krankheit bekomme.

☐ Ich möchte keine Ergebnisse über Krankheitsrisiken in Wahrscheinlichkeiten erhalten.

☐ Weiss nicht.

Q14. Wenn Sie Forschungsergebnisse erhalten wollen würden: Welchen Kommunikationskanal würden Sie bevorzugen?

Kreuzen Sie bitte alle zutreffenden Antworten an.

☐ Brief per Post

☐ Telefonat

☐ E-Mail

☐ Website mit sicherem Login

☐ App

☐ Persönliche Kommunikation / Gespräch

Q15. Wie wichtig wäre Geld oder eine andere materielle Entschädigung für Sie, um Ihre Daten und/oder Proben einer Schweizer öffentlich finanzierten Biobank zur Verfügung zu stellen?

Bitte bewerten Sie auf einer Skala von 1 = nicht wichtig bis 5 = sehr wichtig.

1	2	3	4	5
☐	☐	☐	☐	☐

Zuletzt: Persönliche Informationen

In diesem letzten kurzen Teil bitten wir Sie, einige Informationen über sich selbst anzugeben.

Q16. Wie würden Sie Ihren allgemeinen Gesundheitszustand beschreiben?

☐	☐	☐	☐	☐
Sehr schlecht	Eher schlecht	Mittelmässig	Eher gut	Sehr gut

Q17. Wie alt sind Sie?

☐ 18-24 ☐ 35-44 ☐ 55-64 ☐ 75 oder älter

☐ 25-34 ☐ 45-54 ☐ 65-74

Q18. Was ist Ihr Geschlecht?

☐ Männlich ☐ Divers

☐ Weiblich ☐ keine Angabe

Q19. Haben Sie leibliche Kinder?

☐ Ja ☐ Nein

Q20. Welche Staatsbürgerschaft haben Sie?
Kreuzen Sie bitte alle zutreffenden Antworten an.

☐ Schweiz ☐ Deutschland ☐ Kosovo

☐ Italien ☐ Portugal ☐ Andere/zusätzliche (bitte angeben):

☐ Frankreich ☐ Spanien

____

Q21. Was ist Ihre höchste abgeschlossene Ausbildung?

☐ Keine oder bis 7 Jahre obligatorische Schule

☐ Obligatorische Schule (8 oder 9 Jahre), 10. Schuljahr / Vorlehre oder anderes Brückenangebot

☐ Berufliche Grundbildung (Anlehre, 2- bis 4-jährige Lehre oder Vollzeitberufsschule, Handelsdiplom, Lehrwerkstätte oder ähnliche Ausbildung)

☐ Allgemeinbildende Schule (Gymnasiale Maturität, Berufs- oder Fachmaturität, Lehrkräfte-Seminar, Diplom- oder Fachmittelschule, Verkehrsschule oder ähnliche Ausbildung)

☐ Höhere Fach- und Berufsausbildung mit eidg. Fachausweis, Diplom oder Meisterdiplom, Technikerschule, höhere Fachschule, HTL, HWV, HFG, HFS oder ähnliche Ausbildung

☐ Universität, pädagogische Hochschule, Fachhochschule

Q22. Sind Sie derzeit oder waren Sie jemals im Gesundheitswesen oder in der Gesundheitsforschung tätig?

☐ Ja ☐ Nein

Q23. Würden Sie sich selbst als eine religiöse Person bezeichnen?

☐	☐	☐
Ja, sehr religiös	Ein bisschen religiös	Nein, überhaupt nicht religiös

---

Allgemeine Bemerkungen:

Ende der Umfrage

Vielen Dank, dass Sie sich die Zeit genommen haben an dieser Umfrage teilzunehmen!
Wir schätzen Ihren wertvollen Beitrag!

Bitte senden Sie den ausgefüllten Fragebogen bis zum 23. November 2019 an folgende Adresse. Hierfür können Sie den beiliegenden, vorfrankierten Umschlag verwenden.

Health Ethics and Policy Lab
ETH Zürich
Hottingerstrasse 10
8092 Zürich
