# Supplementary material for: Public willingness to participate in personalized health research and biobanking: A large-scale Swiss survey
Source: PLoS One. 2021 Apr 1;16(4):e0249141. doi: 10.1371/journal.pone.0249141 (PMC8016315; doi:10.1371/journal.pone.0249141)
Supplement: S4 File — (PDF) [file pone.0249141.s006.pdf]

Eidgenössische Technische Hochschule Zürich
Swiss Federal Institute of Technology Zurich

UNIVERSITÄT BERN

Health Ethics & Policy Lab
Chaire de bioéthique, ETH Zurich

Institut de médecine sociale et préventive
Université de Berne

Tel: 044 505 15 13
Email: persmed@ethz.ch

Enquête Suisse

Votre point de vue sur la recherche en santé personnalisée

un sondage d'opinion de l'ETH Zurich, Health Ethics & Policy Lab et l'Université de Berne, Institut de médecine sociale et préventive

Sondage en ligne:

www.persmed.ethz.ch

Mot de passe: XYZ

Introduction

Bienvenue à notre enquête sur la recherche en santé personnalisée!

Imaginez que vous êtes invité à participer à un projet de recherche en santé personnalisée financé par des fonds publics suisses. Ce projet hypothétique recueille des données sur votre santé.

Les types de données recueillies sont des données personnelles (sexe, âge, poids, etc.), des données sur la santé (antécédents médicaux et dossiers médicaux) et des échantillons biologiques (sang, salive, urine, cheveux ou tissus), qui peuvent fournir des renseignements sur votre ADN. Dans ce qui suit, nous les appellerons données et échantillons.

Les données sont stockées dans une biobanque suisse. Il s'agit d'une vaste banque de données gérée par des centres de recherche publics tels que les universités. Les données sont confidentielles et sécurisées.

Cette enquête vous demandera ce que vous pensez du fait de partager vos données personnelles, vos données de santé et vos échantillons biologiques avec une telle biobanque pour la recherche en santé personnalisée. Quelles seraient vos préoccupations ? Quelles seraient vos attentes ?

Pour nous aider à comprendre ce qui est important pour vous, veuillez nous faire part de vos opinions et de votre point de vue sur la mise à disposition de données sur la santé et d'échantillons biologiques pour la recherche. L'enquête n'évalue pas ce que vous savez sur ces sujets. Veuillez répondre à l'enquête honnêtement et au mieux de vos capacités.

Merci d'avoir accepté de répondre au sondage !

Informations supplémentaires :

Pourquoi des personnes atteintes d’une même maladie réagissent-elles si différemment aux traitements ? Pourquoi un médicament fonctionne-t-il très bien chez une personne et pas chez une autre ? Et pourquoi une personne développe-t-elle une maladie et une autre pas ?

La recherche en **santé personnalisée veut** répondre à ces questions afin de **traiter les malades de manière plus précise, personnalisée et efficace à l'avenir.** La recherche en santé personnalisée utilise également des renseignements génétiques et autres renseignements personnels sur des personnes en bonne santé pour en apprendre davantage sur les causes de certaines maladies et sur les risques de les développer.

Pour ce faire, il est important que les chercheurs aient accès au plus grand nombre possible de données sur la santé et d'échantillons de différentes personnes (ayant un état de santé différent) dans les biobanques qui sont constituées.

Comment remplir le questionnaire

- Cochez la case appropriée à côté de la réponse choisie: ☒
- Si vous avez coché la mauvaise case par erreur, remplissez-la complètement avec un stylo à bille, puis cochez la case appropriée.

Partie 1: Votre attitude

Nous aimerions commencer par votre point de vue général sur les questions suivantes:

Q1. Avant aujourd'hui, avez-vous déjà entendu parler de la recherche sur des échantillons biologiques (p. ex. sang, salive, urine, cheveux ou tissus)?

☐ Oui ☐ Non

Q2. Quelle est votre opinion générale à l'égard de la recherche sur des échantillons biologiques humains (comme le sang, la salive, l'urine, les cheveux ou les tissus)?

☐	☐	☐	☐	☐
Très négatif	Plutôt négatif	Indifférent	Plutôt positif	Très positif

---

Q3. Dans quelle mesure pensez-vous qu'il est important pour vous, votre famille ou la société que vous participiez à la recherche en mettant à disposition vos données de santé (antécédents médicaux et dossiers médicaux) et/ou vos échantillons biologiques (sang, salive, urine, cheveux ou tissus) ?

Veuillez noter sur une échelle de 1 = aucune valeur à 5 = valeur élevée.

	1	2	3	4	5	Je ne sais pas
1. Pour moi personnellement	☐	☐	☐	☐	☐	☐
2. Pour ma famille	☐	☐	☐	☐	☐	☐
3. Für die Gesellschaft allgemein	☐	☐	☐	☐	☐	☐

Q4. Participeriez-vous à un projet de recherche qui utiliserait vos données sur la santé et/ou vos échantillons biologiques ?

☐ Oui (passez à la question **4.1**)

☐ Non (passez à la question **5**)

Note : Même si vous avez répondu non à cette question, nous vous demandons de répondre à l'ensemble de ce sondage. Nous aimerions connaître votre opinion sur la meilleure façon d'organiser la recherche avec des données sur la santé et des échantillons biologiques en fonction de votre point de vue.

Q4.1 Quels types de données et d'échantillons seriez-vous prêt à mettre à disposition ?

Veuillez en choisir autant que possible.

☐ Questionnaires sur mon état de santé

☐ Dossiers médicaux me concernant (numériques ou sur papier)

☐ Les antécédents médicaux de ma famille

☐ Mon échantillon de sang

☐ Mes échantillons biologiques que je peux prendre moi-même (par ex. salive, cheveux, urine, écouvillon buccal)

☐ Mes échantillons biologiques qui doivent être prélevés par le personnel médical (p. ex. tissus tumoraux ou de peau)

☐ Mes données sur les réseaux sociaux

☐ Données dérivées d'applications sur ma santé ou mon mode de vie (p. ex. traceur d'exercice, journal alimentaire, fréquence cardiaque, etc.)

Q4.2 Quelles raisons influencent votre participation à cette recherche ?

Veuillez sélectionner jusqu'à 3 plus pertinentes.

☐ Pour accroître les connaissances scientifiques

☐ Être au service de la société et des générations futures

☐ Par sens général du devoir

☐ Pour améliorer l'ensemble des soins de santé

☐ Pour obtenir un avantage personnel en apprenant quelque chose sur ma santé

☐ Pour le bien de ma famille (pour connaître les maladies génétiquement transmissibles)

☐ Autre raison (veuillez préciser) : ______________________________

☐ Je ne sais pas

Q5. Lesquelles des préoccupations suivantes avez-vous ?

Veuillez sélectionner jusqu'à 3 plus pertinentes.

☐ Je m'inquiète de la recherche impliquant des informations sur mes gènes

☐ J'ai peur de ce que je pourrais découvrir sur mes propres risques pour la santé

☐ Je m'inquiète que la confidentialité de mes données ne soit pas préservée

☐ J'ai m'inquiète que quelqu'un pirate et vole mes données

☐ Je m'inquiète que quelqu'un utilise mes données à des fins discriminatoires à mon encontre ou à l'encontre de ma famille (par exemple, par les assurances maladie ou les services financiers)

☐ Je m'inquiète que mes données et échantillons soient utilisés à des fins commerciales ou de marketing plutôt que de recherche

☐ Je refuse que d'autres personnes (comme des entreprises privées ou des chercheurs) profitent financièrement de mes données

☐ J'ai peur des aiguilles ou des procédures pour obtenir les échantillons

☐ Je ne veux pas faire l'effort nécessaire pour participer

☐ Je ne reçois pas d'argent pour cela

☐ Je n'ai pas le temps de contribuer

☐ Je me fiche de la recherche en santé

☐ Aucune raison particulière

☐ Autre (veuillez préciser):

____

____

Q6. Si, hypothétiquement, vous faisiez don de vos données et/ou échantillons à une biobanqueou : quelles informations souhaitez-vous recevoir à l'avance ?

Veuillez sélectionner jusqu'à 3 types d'informations les plus importants pour vous.

☐ Les types exacts de recherche qui seront effectués

☐ Les avantages et les risques potentiels du don de mes données et/ou échantillons

☐ La manière dont les données et les échantillons sont stockés, telle que rendus anonymes, codés ou stockés avec mon nom

☐ Qui a accès à mes données et échantillons

☐ Qui bénéficiera de la recherche

☐ Qui tirera un profit financier de la recherche

☐ Les mesures de sécurité pour préserver la confidentialité et la protection des données et des échantillons

☐ Aucune de ces informations ne m'aiderait à décider

☐ Autre (veuillez préciser) :

____

____

Partie 2: Gestion des données et des échantillons

Dans cette partie, nous aimerions savoir comment vous pensez que les données et les échantillons mis à disposition devraient être gérés.

Q7. Les biobanques aimeraient pouvoir utiliser vos données et échantillons dans de multiples projets de recherche, même à l'avenir. À quelle fréquence aimeriez-vous que la biobanque vous demande la permission d'utiliser vos données et échantillons dans le cadre de projets de recherche ? (Sachant que votre décision peut toujours être modifiée ou votre accord retire.)

- ☐ Ne me demandez qu'une seule fois, lorsque je mets à disposition mes données et/ou échantillons
- ☐ Redemandez-moi pour chaque nouveau projet
- ☐ Cela dépendrait du type de projet à l'étude
- ☐ Je ne sais pas

Q8. Sous quelle forme préféreriez-vous que vos données et échantillons soient conservés?

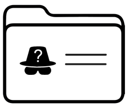

☐ **Anonyme**

<u>Explication :</u>
Les données et les échantillons qui sont anonymisés ne peuvent pas être liés à une personne spécifique sans efforts considérables.
Cela signifie que les identifiants - tels que votre nom, votre adresse, vos photos - sont supprimés de vos données.

<u>Conséquences :</u>
→ Vous ne recevrez que les résultats généraux du projet de recherche.

<u>Risque d'entrave à la vie privée:</u>
Faible
(Les risques pour votre vie privée sont limités, car il est très difficile d'établir un lien entre vos données et vous.)

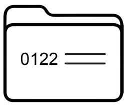

☐ **Codée**

<u>Explication :</u>
Les données et les échantillons sont reliés à une personne spécifique par l'intermédiaire d'un code, lequel est stocké en toute sécurité par les chercheurs. Cela signifie qu'un code ou une clé est nécessaire pour relier les données à vous.

<u>Conséquences :</u>
→ Vous recevez des résultats individuels sur votre statut personnel, en plus des résultats généraux du projet de recherche.

<u>Risque d'entrave à la vie privée</u>*:*
Moyen
(Les risques pour votre vie privée sont limités, car vos données ne peuvent être identifiées qu'à l'aide d'un code sécurisé.)

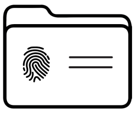

☐ **Identifiable**

<u>Explication</u> :
Les données et les échantillons sont stockés ensemble et étiquetés à votre nom. Ils peuvent facilement être reliés à vous.

<u>Conséquences :</u>
→ Vous recevez des résultats individuels sur votre statut personnel, en plus des résultats généraux du projet de recherche.

<u>Risque d'entrave à la vie privée:</u> Élevé
(Votre vie privée peut être menacée, car il est facile d'identifier vos données.)

Q9. Selon vous, à qui devraient appartenir les données et les échantillons que vous donnez à une biobanque ?

Veuillez choisir une seule réponse.

☐ A moi personnellement

☐ A la biobanque

☐ Au gouvernement suisse

☐ Aux universités impliquées dans la biobanque

☐ Aux chercheurs spécifiques qui font des découvertes

☐ A personne

☐ Autre (veuillez préciser): ____

☐ Je ne sais pas

Partie 3: Gouvernance et partage des données

A présent, nous aimerions savoir comment vous pensez que les données et les échantillons devraient être gérés et partagés.

Q10. Selon vous, qui devrait être responsable de s'assurer que vos données et échantillons sont correctement stockés et gérés ?

Veuillez choisir une réponse.

☐ La biobanque (conseil d'administration)

☐ Un comité indépendant représentant le public (p. ex. les citoyens, les patients, le public)

☐ Un comité d'experts indépendants (p. ex. chercheurs indépendants : scientifiques et cliniciens non associés à la biobanque)

☐ Un comité mixte de citoyens et d'experts

☐ Autre (veuillez préciser): ______________________________

Q11. Imaginez que les personnes et organisations suivantes en Suisse aient accès à vos données et échantillons dans la biobanque. Dans quelle mesure auriez-vous confiance qu'ils garderont vos données et échantillons confidentiels et protégés ?

Veuillez évaluer sur une échelle allant de 1 = aucune confiance à 5 = confiance forte.

	1	2	3	4	5
Mon médecin	☐	☐	☐	☐	☐
Médecins en général	☐	☐	☐	☐	☐
Chercheurs universitaires	☐	☐	☐	☐	☐
Chercheurs d'autres institutions publics	☐	☐	☐	☐	☐
Entreprises pharmaceutiques	☐	☐	☐	☐	☐
Autres entreprises multi-nationales	☐	☐	☐	☐	☐
Autres entreprises suisses	☐	☐	☐	☐	☐
Assurances maladie	☐	☐	☐	☐	☐
Gouvernement suisse	☐	☐	☐	☐	☐

Partie 4: Utilité de recevoir des résultats médicaux personnellement pertinents

Dans cette partie, nous aimerions savoir quels résultats de recherche vous aimeriez recevoir si vous avez mis à disposition des données et des échantillons à une étude.

Q12. Imaginez que vous avez participé à l'étude d'une biobanque et qu'il est possible d'obtenir des résultats de recherche personnels : quels types de résultats de recherche aimeriez-vous recevoir ?

	Oui	Non	Je ne sais pas
1. Renseignements médicaux de base, p. ex. résultats de laboratoire comme les valeurs de sang	☐	☐	☐
2. Comment mon mode de vie influe-t-il sur mon risque de santé (p. ex., tabagisme, poids, etc.)	☐	☐	☐
3. Résultats sur les risques de maladies pour lesquelles il existe des traitements médicales (p. ex. risque de certains types de cancer, certains problèmes cardiaques)	☐	☐	☐
4. Résultats sur les risques de maladies pour lesquelles seules des actions préventives peuvent être entreprises (par exemple, le risque de diabète ou de maladies cardiovasculaires)	☐	☐	☐
5. Résultats sur les risques de maladies pour lesquelles aucun traitement médical n'est disponible, mais qui pourraient avoir une incidence sur mon bien-être ou sur mes décisions de carrière ou planification familiale (p. ex. la maladie d'Alzheimer ou la démence)	☐	☐	☐
6. Résultats de recherche généraux (les résultats ne s'appliquent pas spécifiquement à moi)	☐	☐	☐

Q13. Parfois, les résultats génétiques du développement d'une maladie ne peuvent être exprimés qu'en pourcentage. A quel niveau de probabilité voudriez-vous connaître les résultats ?

Ich möchte Ergebnisse zu meinen Krankheisrisiken erhalten...

☐ Je ne veux recevoir les résultats que si j'aurai **définitivement** la maladie.

☐ Je veux recevoir les résultats lorsqu'il est **très probable** que j'attrape la maladie.

☐ Je veux recevoir les résultats quand c'est **possible, mais pas très probable** que j'attrape la maladie.

☐ Je ne veux pas recevoir de résultats sur les probabilités de maladies.

☐ Je ne sais pas.

Q14. Si vous voulez recevoir des résultats, par quel canal souhaiteriez-vous les recevoir ?

Veuillez cocher toutes les réponses qui s'appliquent.

☐ Lettre par la poste

☐ Appel téléphonique

☐ E-Mail

☐ Site Web avec connexion sécurisée

☐ Application mobile

☐ Communication personnelle (face à face)

Q15. Dans quelle mesure l'argent ou toute autre compensation matérielle serait-il important pour vous pour vous motiver à mettre à disposition vos données et/ou échantillons à une biobanque suisse financée par des fonds publics ?

Veuillez évaluer sur une échelle de 1 = pas important à 5 = très important.

1	2	3	4	5
☐	☐	☐	☐	☐

Dernière partie : Renseignements personnels

Dans cette dernière partie, nous vous demandons de fournir quelques informations vous concernant.

Q16. Comment décririez-vous votre état de santé général?

☐	☐	☐	☐	☐
Très mauvais	Plutôt mauvais	Neutre	Assez bon	Excellent

Q17. Quel âge avez-vous?

☐ 18-24
☐ 25-34
☐ 35-44
☐ 45-54
☐ 55-64
☐ 65-74
☐ 75 ou plus âgé

Q18. Was ist Ihr Geschlecht?

☐ Homme
☐ Femme
☐ Autre
☐ Préfère ne pas le divulguer

Q19. Avez-vous des enfants biologiques?

☐ Oui
☐ Non

Q20. Quelle est votre nationalité (pays)?
Veuillez cocher toutes les réponses qui s'appliquent.

☐ Suisse
☐ Italie
☐ France
☐ Allemagne
☐ Portugal
☐ Espagne
☐ Kosovo
☐ Autre (veuillez préciser) :
____

Q21. Quel est votre plus haut niveau d'éducation?

☐ Aucune ou jusqu'à 7 ans d'études obligatoires
☐ École obligatoire (8 ou 9 ans)
☐ Formation professionnelle de base / apprentissage
☐ École secondaire (Maturité)
☐ Formation professionnelle supérieure / apprentissage ou école technique
☐ Diplôme universitaire (Bachelor / Master / Doctorat)

Q22. Travaillez-vous ou avez-vous déjà travaillé dans le secteur des soins, de la santé, ou de la recherche sur la santé?

☐ Oui ☐ Non

Q23. Vous décririez-vous comme une personne religieuse?

☐	☐	☐
Tout à fait	Un peu	Pas du tout

Remarques générales :

Fin de l'enquête

Merci beaucoup d'avoir pris le temps de répondre à ce sondage!
Nous apprécions grandement votre précieuse contribution!

Veuillez renvoyer le questionnaire complété avant le 23 novembre 2019 à l'adresse suivante. Pour ce faire, vous pouvez utiliser l'enveloppe préaffranchie ci-jointe.

Health Ethics and Policy Lab
ETH Zürich
Hottingerstrasse 10
8092 Zürich
