# Supplementary material for: Public willingness to participate in personalized health research and biobanking: A large-scale Swiss survey
Source: PLoS One. 2021 Apr 1;16(4):e0249141. doi: 10.1371/journal.pone.0249141 (PMC8016315; doi:10.1371/journal.pone.0249141)
Supplement: S5 File — (PDF) [file pone.0249141.s007.pdf]

Eidgenössische Technische Hochschule Zürich
Swiss Federal Institute of Technology Zurich

UNIVERSITÄT BERN

Health Ethics & Policy Lab
Cattedra di bioetica, ETH Zurigo

Istituto di medicina sociale e preventiva
Università di Berna

Tel: 044 505 15 13
Email: persmed@ethz.ch

Sondaggio Svizzero

Il suo punto di vista sulla medicina personalizzata

un sondaggio congiunto di ETH Zurigo, Health Ethics & Policy Lab e Università di Berna, Instituto medicina sociale e preventiva

Sondaggio online

www.persmed.ethz.ch

Password: XYZ

Introduzione

Benvenuti nel nostro sondaggio sulla medicina personalizzata!

Immagini di essere invitata/o da un progetto svizzero di *ricerca sulla medicina personalizzata* finanziato con fondi pubblici. Questo ipotetico progetto raccoglie dati circa la sua salute.

I tipi di dati raccolti sono dati personali (sesso, età, peso ecc.), dati sanitari (anamnesi e cartelle cliniche) e campioni biologici (sangue, saliva, urine, capelli o tessuti), che possono fornire informazioni sul suo DNA. Di seguito li chiameremo dati e campioni.

I dati sono conservati in una biobanca svizzera. Per biobanca si intende una struttura gestita da centri di ricerca pubblici (come ad sempio le università), che raccoglie e conserva dati sanitari e campioni biologici da utilizzare a fini di ricerca in ambito biomedico.
I dati contenuti nella biobanca sono mantenuti confidenziali e sicuri.

Questo sondaggio le porrà il seguente genere di domande: come si sentirebbe a condividere i suoi dati personali, dati sanitari e campioni biologici con una biobanca per una *ricerca sulla medicina personalizzata*? Quali sarebbero le sue preoccupazioni? E quali le sue aspettative?

Per aiutarci a capire ciò che è importante per lei, la preghiamo di condividere le sue opinioni sulla condivisionedi dati sanitari e campioni biologici per la ricerca. L'indagine non sta valutando quanto lei sappia su questi argomenti. La preghiamo di rispondere alle domande del questionario in modo veritiero e al meglio delle sue capacità.

Informazioni aggiuntive sulla *medicina personalizzata*:

Perché alcune malattie colpiscono determinati individui ma ne risparmiano altri? E perché alcuni pazienti reagiscono positivamente ad un determinato farmaco, mentre altri non ne traggono beneficio?

La **ricerca sulla medicina personalizzata vuole** rispondere a queste domande perché in futuro i pazienti possano essere **curati in modo sempre più preciso, personalizzato ed efficace**. A tal fine, la ricerca sulla *medicina personalizzata* si avvale di dati genetici, biologici e sanitari riguardanti sia individui malati che individui sani. Per compiere passi in avanti nell’ambito della medicina personalizzata è dunque necessario che i ricercatori abbiano accesso a questi tipi di dati e possano analizzarli.

Come si compila il questionario?

- Spunta la casella appropriata accanto alla risposta scelta: ☒
- Se avete spuntato per errore la casella sbagliata, riempitela completamente con una penna a sfera e poi spuntate la casella appropriate.

Parte 1: Prospettiva generale

Vorremmo iniziare il questionario sulle seguenti questioni:

Q1. Ad oggi, ha mai sentito parlare di ricerche effettuate utilizzando campioni biologici (ad esempio sangue, saliva, urine, urine, capelli o tessuti)?

☐ Sì ☐ No

Q2. Qual è la sua opinione generale circa la ricerca con campioni biologici umani (come sangue, saliva, urine, capelli o tessuti)?

☐	☐	☐	☐	☐
Molto negativo	Abbastanza negativo	Indifferente	Piuttosto positivo	Molto positivo

---

Q3. Che valore e beneficio avrebbe, secondo lei, partecipare ad una ricerca biomedica fornendo i suoi dati sanitari (anamnesi e cartelle cliniche) e campioni biologici (come sangue, saliva, urine, capelli o tessuti)?

Valuti su una scala da 1 = nessun valore a 5 = valore alto.

	1	2	3	4	5	No lo so
1. Per me personalmente	☐	☐	☐	☐	☐	☐
2. Per la mia famiglia	☐	☐	☐	☐	☐	☐
3. Per la società	☐	☐	☐	☐	☐	☐

Q4. Parteciperebbe ad un progetto di ricerca che utilizza i suoi dati sanitari e/o campioni biologici?

☐ Sì (continuare con la domanda **4.1**)

☐ No (continuare con la domanda **5**)

Nota: anche se ha risposto di no a questa domanda, le chiediamo di completare completamente questo sondaggio. Siamo interessati alla sua opinione su come organizzare al meglio la ricerca con dati sanitari e campioni biologici.

Q4.1 Quali tipi di dati e campioni sarebbe ipoteticamente disposto a fornire?

Selezioni tutte le opzioni per cui è favorevole.

☐ Informazioni sul mio stato di salute

☐ La mia cartella medica (digitale o cartacea)

☐ La mia anamnesi familiare (storia familiare)

☐ Il mio campione di sangue

☐ I miei campioni biologici prelevati da me stesso (ad es. saliva, capelli, urine, tampone buccale)

☐ I miei campioni biologici prelevati da personale medico (ad es. tessuto tumorale, cellule epiteliali)

☐ I miei dati sui social media

☐ I miei dati derivati da applicazioni digitali mobili che riguardano la mia salute o il mio stile di vita (ad es. applicazioni che contano i passi, le calorie ingerite, la frequenza cardiaca, ecc.)

Q4.2 Quali di queste ragioni influenzano il motivo per cui lei parteciperebbe a questa ricerca?

Si prega di selezionarei al massimo 3 ragioni.

☐ Per aumentare le conoscenze scientifiche

☐ Per dare beneficio alla società e alle generazioni future

☐ Per senso generale del dovere

☐ Per migliorare l'assistenza sanitaria globale

☐ Per ottenere un beneficio personale imparando qualcosa sulla mia salute

☐ Per favorire la mia famiglia (per conoscere le malattie genetiche ereditarie)

☐ Per altro motivo (specificare): ____

☐ No lo so

Q5. Quali delle questioni di seguito elencate la preoccuperebbe?

Si prega di selezionarei al massimo 3 ragioni.

☐ Mi preoccupano le ricerche che coinvolgono informazioni sui miei geni

☐ Temo ciò che potrei scoprire sui miei rischi per la salute

☐ Temo che i miei dati non siano mantenuti riservati

☐ Temo che qualcuno possa violare il sistema di protezione e rubare i miei dati

☐ Temo che qualcuno possa utilizzare i miei dati per discriminare me o la mia famiglia (ad esempio da parte di assicurazioni sanitarie o servizi finanziari)

☐ Temo che i miei dati e campioni possano essere utilizzati in modo improprio a fini commerciali o di marketing invece che di ricerca

☐ Non voglio che altri (come aziende private o ricercatori) beneficino finanziariamente dei miei dati

☐ Ho paura degli aghi o delle procedure per ottenere/o campioni

☐ Non voglio fare lo sforzo richiesto per donare/o campioni

☐ Non ricevo denaro per fornire i miei campioni

☐ Non ho tempo per contribuire alla ricerca

☐ Non mi interessa la ricerca sulla salute

☐ Nessun motivo specifico

☐ Altro (specificare):

____

____

Q6. Che lei voglia o meno donare i dati e/o campioni ad un'ipotetica biobanca: quali sarebbero le informazioni piu importanti che vorrebbe avere per prendere questa decisione?

Seleziona fino a 3 tipi di informazioni più importanti per lei.

☐ Vorrei sapere quali ricerche saranno condotte con i miei dati

☐ Vorrei sapere quali sono i potenziali benefici e rischi associati alla donazione dei miei dati e campioni

☐ Vorrei sapere come i miei dati e/o campioni sono memorizzati (specificamente se sono resi anonimi, codificati o memorizzati insieme al mio nome)

☐ Vorrei sapere chi ha accesso ai miei dati e campioni

☐ Vorrei sapere chi trarrà beneficio dalla ricerca

☐ Vorrei sapere chi trarrà profitto finanziario dalla ricerca

☐ Vorrei sapere quali sono le misure di sicurezza utilizzate per mantenere privati e protetti i miei dati e/o campioni

☐ Nessuna di queste informazioni mi aiuterebbe a decidere

☐ Altro (specificare):

____

____

Parte 2: Gestione dei dati e dei campioni

In questa parte vorremmo sapere come pensaa che i dati e/o campioni forniti dovrebbero essere gestiti.

Q7. Supponiamo che la biobanca voglia utilizzare i suoi dati e campioni in molteplici progetti di ricerca, anche in futuro. Con quale frequenza vorrebbe che la biobanca le chiedesse il permesso di utilizzare i suoi dati e/o campioni in progetti di ricerca? (La sua decisione di fornire i dati può sempre essere cambiata o ritirata.)

☐ Solo una volta. Nel momento in cui fornisco i miei dati e/o campioni

☐ Ogni volta che i miei dati e/o campioni servono in un nuovo progetto

☐ Dipenderà dal tipo di progetto in esame

☐ Non sono sicuro

Q8. In quale forma preferirebbe che i suoi dati e campioni fossero conservati?

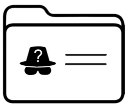

☐ Anonimizzato

Spiegazione:
I dati e/o campioni non possono essere ricollegati ad una persona specifica senza un enorme sforzo. Questo significa che gli identificatori - come il vostro nome, indirizzo, immagini - vengono rimossi dai vostri dati.

Conseguenze:
→ Lei riceverà solo i risultati generali emersi nel progetto di ricerca.

Rischio per la privacy: Basso
(Ci sono rischi limitati per la sua privacy, perché è molto difficile ricollegare i suoi dati a lei stesso.)

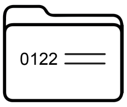

☐ Codificato

Spiegazione:
I dati e/o campioni sono collegati ad una persona specifica tramite un codice che viene conservato in modo sicuro dai ricercatori.
Questo significa che è necessario essere in possesso del codice per ricollegare i dati.

Conseguenze:
→ Oltre ai risultati generali emersi dal progetto di ricerca, potrà ricevere anche i risultati individuali sul suo stato personale di salute.

Rischio per la privacy: Medio
(Ci sono rischi limitati per la vostra privacy, perché i vostri dati potrebbero essere identificati solo se in possesso del codice di sicurezza.)

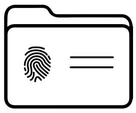

☐ Identificabile

Spiegazione:
I dati e/o campioni sono memorizzati ed etichettati con il vostro nome. Pertanto essi possono essere facilmente ricollegate a lei.

Conseguenze:
→ Oltre ai risultati generali emersi dal progetto di ricerca, potrà ricevere anche i risultati individuali sul suo stato personale di salute.

Rischio per la privacy: Alto
(La vostra privacy può essere a rischio, perché è facile identificare i vostri dati.)

Q9. Chi pensa dovrebbe essere il proprietario dei dati e campioni che lei dona ad una biobanca?

Selezionare una risposta.

☐ Io personalmente

☐ La biobanca

☐ Il governo svizzero

☐ Le università coinvolte con la biobanca

☐ I ricercatori specifici che fanno scoperte

☐ Nessuno

☐ Altro (specificare): ____

☐ Non sono sicuro

Parte 3: Governance e condivisione dei dati

Ora, vorremmo sapere come pensa che i dati e campioni dovrebbero essere governati e condivisi.

Q10. A suo parere, chi dovrebbe essere responsabile della corretta conservazione e gestione dei suoi dati e campioni?

Selezionare una risposta.

☐ La biobanca (consiglio di amministrazione)

☐ Un comitato indipendente che rappresenta i cittadini (ad es. cittadini, pazienti)

☐ Un comitato di esperti indipendenti (ad es. ricercatori indipendenti: scienziati e clinici non associati alla biobanca)

☐ Un comitato misto di pubblico ed esperti

☐ Altro (specificare): ____________________

Q11. Immaginate che le seguenti persone e organizzazioni in Svizzera abbiano accesso ai vostri dati e campioni nella biobanca. In che misura vi fidereste di loro per mantenere i vostri dati e campioni riservati e protetti?

Valutate su una scala da 1 = nessuna fiducia a 5 = forte fiducia.

	1	2	3	4	5
Il mio medico	☐	☐	☐	☐	☐
Medici in generale	☐	☐	☐	☐	☐
Ricercatori in un'università	☐	☐	☐	☐	☐
Ricercatori in altri istituti pubblici	☐	☐	☐	☐	☐
Aziende farmaceutiche	☐	☐	☐	☐	☐
Altre società private globali, a scopo di lucro	☐	☐	☐	☐	☐
Altre società svizzere private svizzere, a scopo di lucro	☐	☐	☐	☐	☐
Assicurazioni sanitarie	☐	☐	☐	☐	☐
Governo svizzero	☐	☐	☐	☐	☐

Parte 4: Utilità di ricevere personalmente risultati medici

In questa parte, vorremmo sapere quali risultati vorrebbe ricevere se donase i suoi dati e campioni per una ricerca.

Q12. Immagini di aver partecipato a una ricerca che include l'analisi dei suoi dati e campioni presenti in una biobanca. Ora lei puo ricevere risultati circa il suo stato di salute emersi da questa ricerca: quali tipi di risultati vorresti ricevere?

	Sì	No	No lo so
1. Risultati medici di base, ad es. risultati di laboratorio come l'emocromo	☐	☐	☐
2. Risultati riguardo come il mio stile di vita influisce sul rischio di contrarre malattie (ad es. fumare, essere sovrappeso, ecc.)	☐	☐	☐
3. Risultati circa il rischio di sviluppare malattie per le quali sono disponibili trattamenti medici (ad es. rischio di sviluppare alcuni tipi di cancro, o alcuni problemi cardiaci)	☐	☐	☐
4. Risultati circa il rischio di sviluppare malattie per le quali possono essere intraprese solo azioni preventive (ad es. rischio di sviluppare il diabete o malattie cardiovascolari)	☐	☐	☐
5. Risultati circa il rischio di sviluppare malattie per le quali non sono disponibili cure mediche ma che potrebbero influire sul mio benessere o sulle decisioni relative alla mia carriera o alla pianificazione familiare (ad es. Alzheimer o demenza)	☐	☐	☐
6. Risultati generali che emergono dalla ricerca (i risultati non si applicano a me in particolare)	☐	☐	☐

Q13. Spesso, i risultati genetici legati allo sviluppo di una malattia possono essere espressi solo in termini di probabilità di sviluppare quella malattia. Quale livello di probabilità di sviluppare una malattia sarebbe sufficiente affinchè lei voglia vedere il risultato?

Selezionare una risposta. Voglio ricevere i miei risultati...

☐ ... quando **c'è certezza** che sviluppi la malattia.

☐ ... quando **è altamente probabile** che io sviluppi la malattia.

☐ ... quando è possibile, ma **non è molto probabile** che io sviluppi la malattia.

☐ Non voglio ricevere alcun risultato sulle probabilità di sviluppare la malattia.

☐ No lo so.

Q14. Nel caso in cui lei volesse ricevere alcuni o tutti i riultati della ricerca, attraverso quale canale di comunicazione vorrebbe riceverli?

Si prega di selezionare tutti quelli che si applicano.

☐ Tramite lettera per posta

☐ Tramite telefonata

☐ Tramite eMail

☐ Tramite sito web con login sicuro

☐ Tramite applicazione mobile (app del cellulare)

☐ Tramite comunicazione personale (faccia a faccia)

Q15. Quanto sarebbe importante per lei ricevere da una biobanca svizzera, finanziata con fondi pubblici, del denaro o qualsiasi altro compenso materiale in cambio dei suoi dati e campioni?

Valutare su una scala da 1 = non importante a 5 = molto importante.

1	2	3	4	5
☐	☐	☐	☐	☐

In conclusione: Informazioni personali

In questa parte finale, le chiediamo di fornire alcune informazioni su di lei.

Q16. Come descriverebbe il suo stato di salute generale?

☐	☐	☐	☐	☐
Ammalato	Di salute precaria	Mediocre	In salute	In ottima salute

Q17. Quanti anni ha?

☐ 18-24
☐ 25-34
☐ 35-44
☐ 45-54
☐ 55-64
☐ 65-74
☐ 75 o più vecchio

Q18. Qual è il suo sesso?

☐ Maschio
☐ Femmina
☐ Altro
☐ Preferiscono non divulgare

Q19. Ha figli biologici?

☐ Ha figli biologici?
☐ No

Q20. Qual è la sua nazionalità?

Si prega di selezionare tutti quelli che si applicano.

☐ svizzero
☐ italiano
☐ francese
☐ tedesco
☐ portoghese
☐ spagnola
☐ kossovara
☐ Altro (specificare:
____

Q21. Qual è il suo più alto livello di istruzione completato?

☐ Nessuno o fino a 7 anni di scuola dell'obbligo
☐ Scuola dell'obbligo scolastico (8 o 9 anni)
☐ Formazione professionale di base / apprendistato
☐ Scuola media superiore (maturità)
☐ Formazione professionale superiore / apprendistato o scuola tecnica
☐ Laurea universitaria (Bachelor / Master / Master / Dottorato di ricerca)

Q22. Lavora o ha mai lavorato nel settore sanitario o della ricerca sanitaria?

☐ Sì ☐ No

Q23. Si descriverebbe come persona religiosa?

☐	☐	☐
Molto	Un po'	No

Se ha ulteriori commenti la preghiamo di elencarli qui:

Fine del sondaggio

Grazie mille per aver dedicato del tempo alla compilazione di questo sondaggio!
Apprezziamo molto il suo prezioso contributo!

Per favore, invii il questionario compilato al seguente indirizzo entro il 23 novembre 2019. A tale scopo utilizzi la busta preaffrancata allegata.

Health Ethics and Policy Lab
ETH Zürich
Hottingerstrasse 10
8092 Zürich
