# Supplementary material for: Public willingness to participate in personalized health research and biobanking: A large-scale Swiss survey
Source: PLoS One. 2021 Apr 1;16(4):e0249141. doi: 10.1371/journal.pone.0249141 (PMC8016315; doi:10.1371/journal.pone.0249141)
Supplement: S6 File — (PDF) [file pone.0249141.s008.pdf]

Eidgenössische Technische Hochschule Zürich
Swiss Federal Institute of Technology Zurich

UNIVERSITÄT BERN

Health Ethics & Policy Lab
Lehrstuhl für Bioethik, ETH Zürich

Institut für Sozial- und Präventivmedizin
Universität Bern

Tel: 044 505 15 13
Email: persmed@ethz.ch

Herr /Frau
Name
Straße
Ort

Zürich, 16. September 2019

Einladung zur Schweizer Umfrage: Ihre Meinung zu personalisierter Gesundheitsforschung

Sehr geehrte/r Herr /Frau XXX

Wir möchten Sie herzlich dazu einladen, an unserer Meinungsumfrage zu personalisierter Gesundheitsforschung teilzunehmen. Die Umfrage wird vom Health Ethics and Policy Lab der ETH Zürich und dem Institut für Sozial- und Präventivmedizin der Universität Bern durchgeführt.

Beantworten der Umfrage:
Die Umfrage dauert ca. 15-20 Minuten. Nachdem Sie die Umfrage beantwortet haben, werden Sie im Zusammenhang mit diesem Projekt nicht mehr kontaktiert.

Bitte rufen Sie den Fragebogen über folgenden Link auf, indem Sie ihn in Ihren Internetbrowser eingeben:

www.persmed.ethz.ch

Verwenden Sie dieses Passwort, um sich anzumelden: **PASSWORT/TOKEN (tbd)**

Was ist das Ziel der Umfrage?
Ziel dieser Umfrage ist es, Einblicke in Ihre allgemeine Einstellung, Bedenken und Erwartungen zu personalisierter Gesundheitsforschung zu gewinnen. Personalisierte Gesundheitsforschung nutzt persönliche Daten, gesundheitsbezogene Daten sowie biologische Proben (zum Beispiel Blut oder Speichel) und speichert diese in Biobanken. Eine Biobank ist eine Datenbank, die von öffentlichen Forschungszentren wie Universitäten betrieben wird und gesundheitsbezogene Daten sowie biologische Proben für die biomedizinische Forschung speichert.

Was ist personalisierte Gesundheitsforschung?

Warum reagieren Menschen mit den gleichen Krankheiten so unterschiedlich auf Behandlungen? Warum wirkt ein Medikament bei dem einen Menschen sehr gut und bei einem anderen nicht? Und warum entwickelt der eine Mensch eine Krankheit und der andere nicht?
Die Forschung im Bereich der personalisierten Medizin will diese Fragen beantworten, um erkrankte Menschen in Zukunft präziser, personalisierter und effektiver zu behandeln. Die personalisierte Gesundheitsforschung nutzt auch genetische und weitere Informationen von gesunden Menschen, um mehr über die Ursachen bestimmter Krankheiten und die Wahrscheinlichkeiten für deren Entstehung zu erfahren. Dabei ist es wichtig, dass Forscher auf möglichst viele verfügbare Gesundheitsdaten verschiedener Personen zugreifen können.

Wie werden die Ergebnisse dieser Umfrage verwendet?

Die Ergebnisse dieser Umfrage liefern Hinweise wie Biobanken für personalisierte Gesundheitsforschung in der Schweiz im Interesse der Bevölkerung aufgebaut und verwaltet werden sollten. Desweiteren werden die Ergebnisse auf wissenschaftlichen Tagungen vorgestellt und in wissenschaftlichen Zeitschriften veröffentlicht. Informationen und Antworten der befragten Personen werden in einer Form gespeichert und ausgewertet, die keine Rückschlüsse auf einzelne Befragte ermöglicht (Anonymität). In dieser Umfrage geht es ausschliesslich um Ihre Meinung zu personalisierter Gesundheitsforschung. Sie werden u.a. gefragt, was Sie über die Teilnahme an hypothetischen Studien denken würden.

Ihre Adresse wurde in einem Zufallsverfahren aus dem Stichprobenregister des Bundesamtes für Statistik (BFS) ermittelt. Die rechtliche Grundlage dafür ist Artikel 13c Abs. 2 Best. c der Statistikerhebungsverordnung (SR 431.012.1).

**Einverständniserklärung**
Mit dem Ausfüllen und Absenden des Fragebogens geben Sie Ihre Einwilligung zur Teilnahme an dieser Umfrage.

Wenn Sie Fragen haben:

Wenn Sie Fragen haben oder an der Umfrage nicht online, sondern per Papierfragebogen teilnehmen möchten, können Sie uns über die E-Mail-Adresse persmed@ethz.ch oder telefonisch unter 044 505 15 13 erreichen.

Wir danken Ihnen herzlich für Ihren wertvollen Beitrag und freuen uns sehr, wenn Sie an dieser Umfrage teilnehmen!

Mit freundlichen Grüssen

Prof. Dr. Effy Vayena
Health Ethics and Policy Lab
ETH Zürich

Prof. Dr. Matthias Egger
Institut für Sozial- und Präventivmedizin
Universität Bern

Weitere Informationen über die Umfrage und Ihre Rechte

Bedingungen für die Teilnahme an der Umfrage:
Um an der Studie teilnehmen zu können, müssen Sie mindestens 18 Jahre alt und in der Schweiz wohnhaft sein. Die Adressen stammen aus dem Einwohnerregister des Bundesamtes für Statistik.

Widerrufsrecht:
Sie haben das Recht, Ihre Teilnahme an der Umfrage jederzeit, ohne Angabe von Gründen, und ohne Konsequenzen zu widerrufen.

Vor- und Nachteile der Teilnahme:
Mit dem Ausfüllen der Umfrage sind keine Vor- oder Nachteile für Sie verbunden. Es wird keine Entschädigung für das Ausfüllen der Umfrage angeboten. Die Umfrage verfolgt kein kommerzielles, sondern ein rein wissenschaftliches und soziales Ziel. Das Ausfüllen dieser Umfrage bedeutet nicht, Ihre Daten und Proben einer nationalen, öffentlich finanzierten Biobank zur Verfügung zu stellen.

Datenschutz:
Ihre Antworten werden sicher gespeichert und anonymisiert. Nur die zuständigen Forscher und/oder die Mitglieder der ETH Ethikkommission haben Zugang zu den Originalantworten unter strikter Beachtung der Vertraulichkeitsregeln.

Finanzierungsinformationen und Bewilligung Ethikkommission:
Diese Umfrage wird mit internen Ressourcen des Health Ethics and Policy Labs der ETH Zürich und des Instituts für Sozial- und Präventivmedizin der Universität Bern finanziert. Die Studie wurde von der Ethikkommission der ETH Zürich (EK 2018-N-66) genehmigt.
