# Supplementary material for: Public willingness to participate in personalized health research and biobanking: A large-scale Swiss survey
Source: PLoS One. 2021 Apr 1;16(4):e0249141. doi: 10.1371/journal.pone.0249141 (PMC8016315; doi:10.1371/journal.pone.0249141)
Supplement: S7 File — (PDF) [file pone.0249141.s009.pdf]

ETH
Eidgenössische Technische Hochschule Zürich
Swiss Federal Institute of Technology Zurich

UNIVERSITÄT BERN

Health Ethics and Policy Lab
Chaire de bioéthique, ETH Zurich

Institut de médecine sociale et préventive
Université de Berne

Tel: 044 505 15 13
Email: persmed@ethz.ch

M./Mme/Mlle
Nom
Rue
Commune

Zurich, 16 septembre 2019

Invitation à une enquête suisse : votre point de vue sur la recherche en santé personnalisée

Cher Monsieur, chère Madame,

Nous souhaiterions vous inviter à participer à un sondage sur la recherche en santé personnalisée. L'enquête est menée par le Health Ethics and Policy Lab de l'ETH Zurich et l'Institut de médecine sociale et préventive de l'Université de Berne.

Au sujet du questionnaire
Vous aurez besoin d'environ 15 à 20 minutes pour répondre au sondage. Après avoir rempli le questionnaire, nous ne vous recontacterons plus dans le cadre de ce projet.

Veuillez accéder au questionnaire via le lien suivant, en le copiant dans votre navigateur Internet:

www.persmed.ethz.ch

et utilisez le mot de passe suivant pour vous connecter: **MOT DE PASSE/TOKEN**

Quel est le but de l'enquête ?
L'objectif de cette enquête est de connaître votre attitude générale, vos croyances, vos préoccupations et vos attentes en ce qui concerne votre participation éventuelle à des projets de recherche en santé personnalisés financés par les fonds publics suisses. Ces projets inclueraient la collecte de données personnelles, de données sur la santé et/ou d'échantillons biologiques dans une biobanque. Une biobanque est une banque de données, gérée par des centres de recherche publics tels que les universités, qui stockent des données sur la santé et des échantillons biologiques à des fins de recherche.

Qu'est-ce que la recherche en santé personnalisée ?

Pourquoi les personnes atteintes des mêmes maladies réagissent-elles différemment aux traitements proposes ? Pourquoi un médicament fonctionne-t-il très bien chez une personne et pas chez une autre ? Et pourquoi une personne développe-t-elle une maladie et une autre pas ?
La recherche en santé personnalisée veut répondre à ces questions afin de traiter les malades de manière plus précise, personnalisée et efficace à l'avenir. La recherche en santé personnalisée utilise également des informations génétiques et autres au sujet de personnes en bonne santé afin d'en savoir plus sur certaines maladies et sur les risques de les développer.
Pour ce faire, il est important que les chercheurs aient accès au plus grand nombre possible de données disponibles sur la santé de différentes personnes (ayant un état de santé différent).

Comment les résultats de cette enquête seront-ils utilises ?

Les résultats de cette enquête indiqueront comment les résidents suisses pensent que les bases de données sur la santé pour la recherche en santé personnalisée devraient être construites et gérées. Les résultats généraux éclaireront l'élaboration des politiques relatives aux biobanques, seront présentés lors de réunions scientifiques et publiés dans des revues scientifiques. Votre identité ne sera jamais dévoilée.
Ce sondage n'est qu'un sondage d'opinion. Certaines questions vous demandent ce que vous pensez du fait de participer à des études hypothétiques.

Votre adresse provient d'un échantillon aléatoire du registre de l'Office fédéral de la statistique (OFS). La base juridique en est l'article 13c, paragraphe 2, de l'Ordonnance sur les enquêtes statistiques (RS 431.012.1).

**Déclaration de consentement**
En remplissant et en retournant ce questionnaire, vous donnez votre consentement éclairé à participer à cette recherche.

Si vous avez des questions

Si vous avez des questions ou si vous souhaitez participer à l'enquête en utilisant un questionnaire papier au lieu de la version en ligne, vous pouvez nous contacter à persmed@ethz.ch ou par téléphone à 044 505 15 13.

Nous vous remercions d'avance pour votre participation à ce sondage.

Meilleures salutations,

Prof. Dr. Effy Vayena
Health Ethics and Policy Lab
ETH Zurich

Prof. Matthias Egger
Institut de médecine sociale et préventive
Université de Berne

Informations complémentaires sur l'enquête et sur vos droits

Conditions de participation à l'enquête

Pour participer à l'enquête, vous devez être âgé d'au moins 18 ans et résider en Suisse. Les adresses ont été fournies par le registre de la population de l'Office fédéral de la statistique.

Droit de rétractation

Vous avez le droit de vous retirer de l'enquête à tout moment, sans indication de motifs et sans conséquences.

Avantages et inconvénients pour les participants

Il n'y a aucun avantage ou inconvénient à remplir le questionnaire. De plus, aucune compensation ne sera offerte pour participer à ce sondage. L'enquête ne poursuit aucun objectif commercial, mais bien un objectif purement scientifique et social. Remplir ce questionnaire ne signifie pas rejoindre une biobanque nationale financée par l'État qui recueille et stocke des données et des échantillons à des fins de recherche.

Protection des données

Vos réponses seront conservées en toute sécurité et communiquées sous forme anonyme. Seuls les enquêteurs responsables et/ou les membres de la commission d'éthique de l'ETH Zurich auront accès aux réponses originales, dans le strict respect des règles de confidentialité.

Information sur le financement et approbation éthique

Cette enquête est financée par des ressources internes du Health Ethics and Policy Lab de l'ETH Zurich et de l'Institut de médecine sociale et préventive (ISPM) de l'Université de Berne. L'étude a été approuvée par la commission d'éthique de l'ETH Zurich (EK 2018-N-66).
