# Supplementary material for: Public willingness to participate in personalized health research and biobanking: A large-scale Swiss survey
Source: PLoS One. 2021 Apr 1;16(4):e0249141. doi: 10.1371/journal.pone.0249141 (PMC8016315; doi:10.1371/journal.pone.0249141)
Supplement: S8 File — (PDF) [file pone.0249141.s010.pdf]

Eidgenössische Technische Hochschule Zürich
Swiss Federal Institute of Technology Zurich

UNIVERSITÄT BERN

Health Ethics and Policy Lab
Cattedra di bioetica, ETH Zurigo

Istituto di medicina sociale e preventiva
Università di Berna

Tel: 044 505 15 13
Email: persmed@ethz.ch

Signor / Signora
Nome
Via
Luogo

Zurigo, 16 Settembre 2019

Invito a partecipare al sondaggio: Il suo punto di vista sulla *medicina personalizzata*

Gentile Signor / Signora XXX

La invitiamo a partecipare a un sondaggio volto a comprendere il suo punto di vista a proposito della ricerca sulla medicina personalizzata. L'indagine è coordinata e condotta dal Centro di Bioetica (Health Ethics and Policy Lab) del Politecnico Federale di Zurigo e dall'Istituto di Medicina Sociale e Preventiva dell'Università di Berna.

Compilazione del questionario:
Al fine di realizzare tale sondaggio, la invitiamo a compilare un questionario online. Avrà bisogno di circa 15-20 minuti per completare il questionario. Dopo aver compilato il questionario, non verrà più contattata/o in relazione a questo progetto.

La invitiamo ad accedere al questionario tramite il seguente link, digitandolo nel suo browser Internet:

www.persmed.ethz.ch

Dovrà inserire questa *password* per partecipare al questionario: **PASSWORD/TOKEN (tbd)**

Qual è l'obiettivo del sondaggio?
L'obiettivo di questo sondaggio è quello di conoscere il suo punto di vista, le sue convinzioni, preoccupazioni e aspettative riguardo alla partecipazione a progetti di ricerca nell'ambito della medicina personalizzata finanziati con fondi pubblici di provenienza Svizzera. Tali progetti comprendono la raccolta di dati personali, dati sanitari e/o campioni biologici conservati presso una biobanca. Una biobanca è una struttura gestita da centri di ricerca pubblici (come ad esempio le università), che raccoglie e conserva dati sanitari e campioni biologici da utilizzare a fini di ricerca in ambito biomedico.

Che cos'è la *medicina personalizzata*?
Perché alcune malattie colpiscono determinati individui ma ne risparmiano altri? E perché alcuni pazienti reagiscono positivamente ad un determinato farmaco, mentre altri non ne traggono beneficio? La ricerca sulla medicina personalizzata vuole rispondere a queste domande perché in futuro i pazienti possano essere curati in modo sempre più preciso, personalizzato ed efficace. A tal fine, la ricerca sulla medicina personalizzata si avvale di dati genetici, biologici e sanitari riguardanti sia individui malati che individui sani. Per compiere passi in avanti nell'ambito della medicina personalizzata è dunque necessario che i ricercatori abbiano accesso a questi tipi di dati e possano analizzarli.

Come saranno utilizzati i risultati di questa indagine?
I risultati del presente sondaggio forniranno indicazioni circa il punto di vista dei residenti svizzeri in merito alla costruzione e gestione di biobanche per la medicina personalizzata. I risultati ottenuti saranno utili per lo sviluppo futuro di biobanche per la ricerca, saranno presentati a convegni scientifici e verranno inclusi in pubblicazioni scientifiche. I risultati saranno sempre presentati in forma aggregata, pertanto la sua identità non sarà mai rivelata né condivisa.

Questo sondaggio è un sondaggio d'opinione. In alcune domande le verrà chiesto di esprimere la sua opinione circa scenari ipotetici.

Il suo nominativo e il suo indirizzo fanno parte di un campione casuale estratto dal registro anagrafico dell'Ufficio Federale di Statistica (UST) appositamente per questo studio. La base giuridica di questo studio è costituita dall'articolo 13c, paragrafo 2, dell'ordinanza sulle rilevazioni statistiche (RS 431.012.1).

**Dichiarazione di consenso**
Completando e restituendo il questionario, sta fornendo il suo consenso informato a prendere parte a questa ricerca.

In caso avesse domande:
Se ha domande o desidera partecipare al sondaggio utilizzando una versione cartacea del questionario, può contattarci all'indirizzo persmed@ethz.ch o per via telefonica al numero 044 505 15 13.

La ringraziamo per la cortese attenzione e per la sua collaborazione qualora decidesse di partecipare a questo sondaggio!

Cordiali Saluti,

Prof. Dr. Effy Vayena
Health Ethics and Policy Lab
ETH Zurigo

Prof. Dr. Matthias Egger
Istituto di medicina sociale e preventiva
Università di Berna

Ulteriori informazioni circa il sondaggio e i suoi diritti

Condizioni per la partecipazione all'indagine:

Per partecipare al sondaggio è necessario avere almeno 18 anni e risiedere in Svizzera. Gli indirizzi dei partecipanti sono stati selezionati riferendosi al registro anagrafico dell'Ufficio Federale di Statistica.

Diritto di revoca:

Lei ha il diritto di ritirarsi dall'indagine in qualsiasi momento senza specificarne i motivi e senza conseguenze di alcun tipo.

Vantaggi e svantaggi per i partecipanti:

Non ci sono vantaggi o svantaggi associati alla compilazione del questionario. Inoltre, non le sarà offerto alcun compenso per la compilazione del questionario. L'indagine non ha finalità di natura commerciale. Compilare questo questionario NON SIGNIFICA fornire i propri dati ad una biobanca che raccoglie e conserva dati e campioni per scopi di ricerca.

Protezione dei dati:

Le vostre risposte saranno conservate in modo sicuro e successivamente anonimizzate. Solo i ricercatori responsabili dell'indagine e/o i membri del comitato etico del Politecnico Federale di Zurigo avranno accesso alle risposte originali e potranno avervi accesso, qualora necessario, solo ne rigoroso rispetto di regole di riservatezza e in osservanza alle disposizioni vigenti in materia di protezione dei dati personali.

Informazioni sul finanziamento e approvazione etica:

Questa indagine è finanziata con risorse interne del Centro di Bioetica (Health Ethics and Policy Lab) del Politecnico Federale di Zurigo e dell'Istituto di Medicina Sociale e Preventiva (ISPM) dell'Università di Berna. Lo studio è stato approvato dalla Commissione etica del Politecnico Federale di Zurigo (EK 2018-N-66).
