# Supplementary material for: Public willingness to participate in personalized health research and biobanking: A large-scale Swiss survey
Source: PLoS One. 2021 Apr 1;16(4):e0249141. doi: 10.1371/journal.pone.0249141 (PMC8016315; doi:10.1371/journal.pone.0249141)
Supplement: S9 File — (PDF) [file pone.0249141.s011.pdf]

ETH
Eidgenössische Technische Hochschule Zürich
Swiss Federal Institute of Technology Zurich

b UNIVERSITÄT BERN

Health Ethics & Policy Lab
Lehrstuhl für Bioethik, ETH Zürich

Institut für Sozial- und Präventivmedizin
Universität Bern

Tel: 044 505 15 13
Email: persmed@ethz.ch

Herr /Frau
Name
Straße
Ort

Zürich, 7. Oktober 2019

Ihre Meinung zu personalisierter Gesundheitsforschung: Erinnerung zur Teilnahme an der Umfrage

Sehr geehrte/r Herr /Frau XXX

Vor drei Wochen haben wir Sie zu einer Meinungsumfrage der ETH Zürich und Universität Bern zum Thema *Personalisierte Gesundheitsforschung* eingeladen. Wir erlauben uns, Sie noch einmal höflich um Ihre Teilnahme an dieser Umfrage zu bitten. Das Ausfüllen dauert nur etwa 15 bis 20 Minuten. Falls Sie zwischenzeitlich bereits teilgenommen haben, bitten wir Sie, dieses Schreiben nicht weiter zu beachten und danken Ihnen sehr herzlich für Ihr Mitwirken.

Wir haben Sie und die anderen Befragten per Zufall aus der Schweizer Bevölkerung ausgewählt. Hierdurch wollen wir sicher stellen, dass alle Meinungen zum Thema *Personalisierte Gesundheitsfoschung* Gehör finden. Ihre Antworten werten wir anonym aus, d.h. es sind keine Rückschlüsse auf Sie persönlich möglich. Wir verfolgen keine kommerziellen, sondern rein wissenschaftliche und gesellschaftliche Ziele. Abgesehen von der ETH Zürich und Universität Bern sind keine weiteren Kooperationspartner involviert.

Zur Umfrage gelanden Sie über folgenden Link: **www.persmed.ethz.ch**

Passwort: **PASSWORT/TOKEN**

Wenn Sie Fragen haben, können Sie uns über die E-Mail-Adresse persmed@ethz.ch oder telefonisch unter 044 505 15 13 erreichen.

Wir hoffen, dass wir auf Ihre Teilnahme zählen dürfen und danken Ihnen bereits jetzt für Ihren wertvollen Beitrag!

Mit freundlichen Grüssen

Prof. Dr. Effy Vayena
Health Ethics and Policy Lab
ETH Zürich

Prof. Dr. Matthias Egger
Institut für Sozial- und Präventivmedizin
Universität Bern
