# Supplementary material for: Public willingness to participate in personalized health research and biobanking: A large-scale Swiss survey
Source: PLoS One. 2021 Apr 1;16(4):e0249141. doi: 10.1371/journal.pone.0249141 (PMC8016315; doi:10.1371/journal.pone.0249141)
Supplement: S10 File — (PDF) [file pone.0249141.s012.pdf]

Eidgenössische Technische Hochschule Zürich
Swiss Federal Institute of Technology Zurich

UNIVERSITÄT
BERN

Health Ethics and Policy Lab
Chaire de bioéthique, ETH Zurich

Institut de médecine sociale et préventive
Université de Berne

Tel: 044 505 15 13
Email: persmed@ethz.ch

M./Mme/Mlle
Nom
Rue
Commune

Zurich, le 07 octobre 2019

Votre point de vue sur la recherche en santé personnalisée : Rappel de participation à l'enquête

Cher Monsieur, chère Madame,

Il y a trois semaines, nous vous avons invité à un sondage d'opinion sur la recherche personnalisée en santé mené par l'ETH Zurich et l'Université de Berne. Nous nous permettons de vous demander à nouveau poliment de participer à cette enquête. Celle-ci ne dure qu'entre 15 à 20 minutes.
Si vous y avez participé entre-temps, nous vous prions de ne pas prêter attention à cette lettre et nous vous remercions pour votre coopération.

Vous avez été selectionné au hasard parmi la population suisse. De cette manière, nous voulons nous assurer que toutes les opinions sur le sujet de la recherche en santé personnalisée soient entendues. Nous évaluons vos réponses de manière anonyme, c'est-à-dire qu'aucune conclusion ne peut être tirée sur vous personnellement. Nous ne poursuivons pas d'objectifs commerciaux, mais purement scientifiques et sociaux. En dehors de l'ETH Zurich et de l'Université de Berne, aucun autre partenaire coopératif n'est impliqué.

Le lien suivant vous mènera au sondage : **www.persmed.ethz.ch**

Mot de passe : **PASSWORD/TOKEN**

Si vous avez des questions, vous pouvez nous contacter par e-mail à persmed@ethz.ch ou par téléphone au 044 505 15 13.

Nous espérons pouvoir compter sur votre participation et vous remercions dès maintenant pour votre précieuse contribution !

Meilleures salutations,

Prof. Dr. Effy Vayena
Health Ethics and Policy Lab
ETH Zurich

Prof. Matthias Egger
Institut de médecine sociale et préventive
Université de Berne
