# Supplementary material for: Public willingness to participate in personalized health research and biobanking: A large-scale Swiss survey
Source: PLoS One. 2021 Apr 1;16(4):e0249141. doi: 10.1371/journal.pone.0249141 (PMC8016315; doi:10.1371/journal.pone.0249141)
Supplement: S11 File — (PDF) [file pone.0249141.s013.pdf]

ETH
Eidgenössische Technische Hochschule Zürich
Swiss Federal Institute of Technology Zurich

UNIVERSITÄT BERN

Health Ethics and Policy Lab
Cattedra di bioetica, ETH Zurigo

Istituto di medicina sociale e preventiva
Università di Berna

Tel: 044 505 15 13
Email: persmed@ethz.ch

Signor / Signora
Nome
Via
Luogo

Zurigo, 07 Ottobre 2019

Il suo punto di vista sulla *medicina personalizzata*: Promemoria per partecipare al sondaggio

Gentile Signor / Signora XXX

Tre settimane fa la abbiamo invitata/o a partecipare ad un sondaggio d'opinione sulla medicina personalizzata condotta dal Politecnico di Zurigo e dall'Università di Berna. Ci permettiamo di invitarla gentilmente a completare il questionario. Ci vogliono solo dai 15 ai 20 minuti per completarlo.
Se ha già completato il questionario, non serve che lei presti ulteriore attenzione a questa lettera e la ringraziamo per la sua collaborazione.

Abbiamo selezionato lei e gli altri intervistati in modo casuale tra la popolazione svizzera. In questo modo, vogliamo garantire che tutte le opinioni sul tema della medicina personalizzata siano raccolte. Analizzeremo le sue risposte in forma anonima; pertanto, non ci sarà possibile trarre conclusioni personali su di lei. Non perseguiamo obiettivi commerciali, ma puramente scientifici e sociali. Oltre al Politecnico di Zurigo e all'Università di Berna, non sono coinvolti altri partner che cooperano a questo progetto.

Il seguente link le permetterà di accedere al sondaggio: **/www.persmed.ethz.ch**

Password: **PASSWORD/TOKEN**

In caso di ulteriori domande, può contattarci via e-mail all'indirizzo persmed@ethz.ch o per telefono al numero 044 505 15 15 13.

Speriamo di poter contare sulla sua partecipazione e la ringraziamo in anticipo per il suo prezioso contributo!

Cordiali Saluti,

Prof. Dr. Effy Vayena
Health Ethics and Policy Lab
ETH Zurigo

Prof. Dr. Matthias Egger
Istituto di medicina sociale e preventiva
Università di Berna
