# Supplementary material for: Public willingness to participate in personalized health research and biobanking: A large-scale Swiss survey
Source: PLoS One. 2021 Apr 1;16(4):e0249141. doi: 10.1371/journal.pone.0249141 (PMC8016315; doi:10.1371/journal.pone.0249141)
Supplement: S12 File — (PDF) [file pone.0249141.s014.pdf]

ETH
Eidgenössische Technische Hochschule Zürich
Swiss Federal Institute of Technology Zurich

UNIVERSITÄT BERN

Health Ethics & Policy Lab
Lehrstuhl für Bioethik, ETH Zürich

Institut für Sozial- und Präventivmedizin
Universität Bern

Tel: 044 505 15 13
E-mail: persmed@ethz.ch

Herr /Frau
Name
Straße
Ort

Zürich, 29. Oktober 2019

Ihre Meinung zu personalisierter Gesundheitsforschung: Erinnerung zur Teilnahme an unserer Umfrage

Sehr geehrte/r Herr /Frau XXX

Im September haben wir Sie zu einer Meinungsumfrage zum Thema *Personalisierte Gesundheitsforschung* eingeladen. Wir möchten Sie noch einmal herzlich bitten, an dieser Umfrage teilzunehmen. Das Ausfüllen des Fragebogens dauert nur etwa 15 bis 20 Minuten. Falls Sie diesen zwischenzeitlich bereits ausgefüllt haben, danken wir Ihnen für Ihr Mitwirken.

Um Ihnen die Teilnahme zu erleichtern, finden Sie den Fragebogen in Papierform beigefügt. Wir sind Ihnen sehr dankbar, wenn Sie diesen ausfüllen und mit dem beiliegenden, vorfrankierten Antwortcouvert bis zum 23. November 2019 an uns zurückschicken. Selbstverständlich können Sie die Umfrage auch weiterhin online ausfüllen unter:

www.persmed.ethz.ch Ihr Passwort ist: **PASSWORT/TOKEN**

Sie wurden per Zufall aus der Schweizer Bevölkerung ausgewählt. Ihre Antworten werten wir anonym aus, so dass keine Rückschlüsse auf Sie persönlich möglich sind. Wir verfolgen keine kommerziellen, sondern rein wissenschaftliche und gesellschaftliche Ziele. Abgesehen von der ETH Zürich und Universität Bern sind keine weiteren Kooperationspartner involviert.

Bei allfälligen Fragen, erreichen Sie uns über die E-Mail-Adresse persmed@ethz.ch oder telefonisch unter 044 505 15 13.

Wir hoffen auf Ihre Beteiligung und danken Ihnen bereits jetzt herzlich für Ihren wertvollen Beitrag!

Mit freundlichen Grüssen

Prof. Dr. Effy Vayena
Health Ethics and Policy Lab
ETH Zürich

Prof. Dr. Matthias Egger
Institut für Sozial- und Präventivmedizin
Universität Bern
