# Supplementary material for: Public willingness to participate in personalized health research and biobanking: A large-scale Swiss survey
Source: PLoS One. 2021 Apr 1;16(4):e0249141. doi: 10.1371/journal.pone.0249141 (PMC8016315; doi:10.1371/journal.pone.0249141)
Supplement: S13 File — (PDF) [file pone.0249141.s015.pdf]

ETH
Eidgenössische Technische Hochschule Zürich
Swiss Federal Institute of Technology Zurich

UNIVERSITÄT BERN

Health Ethics and Policy Lab
Chaire de bioéthique, ETH Zurich

Institut de médecine sociale et préventive
Université de Berne

Tel: 044 505 15 13
Email: persmed@ethz.ch

Cher Monsieur, chère Madame,

M./Mme/Mlle
Nom
Rue
Commune

Zurich, le 29 octobre 2019

**Votre point de vue sur la recherche en santé personnalisée : Rappel de participation à l'enquête**

En septembre, nous vous avons invité à un sondage d'opinion sur la recherche personnalisée en santé. Nous aimerions vous demander encore une fois de participer à cette enquête. Remplir le questionnaire ne prend que 15 à 20 minutes environ. Si vous l'avez déjà fait entre-temps, nous vous remercions pour votre contribution et nous vous prions d'ignorer ce rappel.

Pour faciliter votre participation, vous trouverez ci-joint le questionnaire papier. Nous vous remercions de bien vouloir remplir ce formulaire et de nous le renvoyer avant le 23 novembre 2019 en utilisant l'enveloppe réponse pré-affranchie ci-jointe. Vous pouvez, bien sûr, continuer à remplir le sondage en ligne à l'adresse suivante :

**www.persmed.ethz.ch** Votre mot de passe est : **PASSWORT**

Vous avez été selectionné au hasard parmi la population suisse. Nous évaluons vos réponses de manière anonyme, c'est-à-dire qu'aucune conclusion ne peut être tirée sur vous personnellement. Nous ne poursuivons pas d'objectifs commerciaux, mais purement scientifiques et sociaux. En dehors de l'ETH Zurich et de l'Université de Berne, aucun autre partenaire n'est impliqué.

Si vous avez des questions, vous pouvez nous contacter par e-mail à persmed@ethz.ch ou par téléphone au 044 505 15 13.

Nous espérons pouvoir compter sur votre participation et vous remercions dès maintenant pour votre précieuse contribution !

Meilleures salutations,

Prof. Dr. Effy Vayena
Health Ethics and Policy Lab
ETH Zurich

Prof. Matthias Egger
Institut de médecine sociale et préventive
Université de Berne
