# Supplementary material for: Public willingness to participate in personalized health research and biobanking: A large-scale Swiss survey
Source: PLoS One. 2021 Apr 1;16(4):e0249141. doi: 10.1371/journal.pone.0249141 (PMC8016315; doi:10.1371/journal.pone.0249141)
Supplement: S14 File — (PDF) [file pone.0249141.s016.pdf]

ETH
Eidgenössische Technische Hochschule Zürich
Swiss Federal Institute of Technology Zurich

UNIVERSITÄT BERN

Health Ethics and Policy Lab
Cattedra di bioetica, ETH Zurigo

Istituto di medicina sociale e preventiva
Università di Berna

Tel: 044 505 15 13
Email: persmed@ethz.ch

Signor / Signora
Nome
Via
Luogo

Zurigo, 29 Ottobre 2019

Il suo punto di vista sulla *medicina personalizzata*: Promemoria per partecipare al sondaggio

Egregio Signore / Gentile Signora XXX

A Settembre l'abbiamo invitata a partecipare ad un sondaggio d'opinione sulla medicina personalizzata. Vorremmo chiederle di nuovo di partecipare a questo sondaggio. Ci vogliono solo dai 15 ai 20 minuti per completarlo.
Se nel frattempo avesse già completato il questionario, la ringraziamo per la sua collaborazione.

Per facilitare la sua partecipazione, il questionario è allegato in forma cartacea. Le saremmo molto grati se potesse completarlo e restituirlo entro il 23 novembre 2019 utilizzando la busta di risposta preaffrancata allegata. Naturalmente, è ancora possibile completare il sondaggio online all'indirizzo:

www.persmed.ethz.ch La sua password è: **PASSWORT/TOKEN**

Il suo nominativo è stato scelto a caso all'interno della popolazione svizzera. Analizzeremo le sue risposte in forma anonima; pertanto, non ci sarà possibile trarre conclusioni personali su di lei. Non perseguiamo obiettivi commerciali, ma puramente scientifici e sociali. Oltre al Politecnico di Zurigo e all'Università di Berna, non sono coinvolti altri partner che cooperano a questo progetto.

In caso di ulteriori domande, può contattarci via e-mail all'indirizzo persmed@ethz.ch, o per telefono al numero 044 505 15 13.

Speriamo di poter contare sulla sua partecipazione e la ringraziamo in anticipo per il suo prezioso contributo!

Cordiali Saluti,

Prof. Dr. Effy Vayena
Health Ethics and Policy Lab
ETH Zurigo

Prof. Dr. Matthias Egger
Istituto di medicina sociale e preventiva
Università di Berna
